# Transforming growth factor-β pathway activity in glioblastoma

**DOI:** 10.18632/oncotarget.3467

**Published:** 2015-02-28

**Authors:** Karl Frei, Dorothee Gramatzki, Isabel Tritschler, Judith Johanna Schroeder, Larisa Espinoza, Elisabeth Jane Rushing, Michael Weller

**Affiliations:** ^1^ Department of Neurosurgery, University Hospital Zurich, Zurich, Switzerland; ^2^ Laboratory for Molecular Neuro-Oncology, Department of Neurology, University Hospital Zurich, Zurich, Switzerland; ^3^ Department of Neuropathology, University Hospital Zurich, Zurich, Switzerland; ^4^ Neuroscience Center Zurich, University of Zurich, Zurich, Switzerland

**Keywords:** TGF-β, PDGF-B, PAI-1, glioblastoma, biomarker

## Abstract

Transforming growth factor (TGF)-β is a central molecule maintaining the malignant phenotype of glioblastoma. Anti-TGF-β strategies are currently being explored in early clinical trials. Yet, there is little contemporary data on the differential expression of TGF-β isoforms at the mRNA and protein level or TGF-β/Smad pathway activity in glioblastomas *in vivo*.

Here we studied 64 newly diagnosed and 16 recurrent glioblastomas for the expression of TGF-β_1-3_, platelet-derived growth factor (PDGF)-B, and plasminogen activator inhibitor (PAI)-1 mRNA by RT-PCR and for the levels of TGF-β_1-3_ protein, phosphorylated Smad2 (pSmad2), pSmad1/5/8 and PAI-1 by immunohistochemistry.

Among the TGF-β isoforms, TGF-β_1_ mRNA was the most, whereas TGF-β_3_ mRNA was the least abundant. TGF-β_1-3_ mRNA expression was strongly correlated, as was the expression of TGF-β_1-3_ mRNA, and of the TGF-β_1-3_ target genes, PDGF-B and PAI-1. TGF-β_2_ and TGF-β_3_ protein levels correlated well, whereas the comparison of the other TGF-βisoforms did not. Positive correlation was also observed between TGF-β_1_ and pSmad1/5/8 and between pSmad2 and pSmad1/5/8. Survival analyses indicated that a group of patients with high expression levels of TGF-β_2_ mRNA or pSmad1/5/8 protein have inferior outcome.

We thus provide potential biomarkers for patient stratification in clinical trials of anti-TGF-β therapies in glioblastoma.

## INTRODUCTION

Glioblastoma is the most common and lethal primary brain tumor. Standard of care includes surgery followed by radiotherapy plus concurrent and maintenance chemotherapy with the oral methylating agent, temozolomide (TMZ/RT→TMZ) [1]. Numerous efforts using molecularly targeted therapeutics have not significantly changed the near uniform lethality of this disease. Factors influencing malignancy and progression in gliomas include the transforming growth factor-β (TGF-β) signaling pathway, which modulates invasiveness, angiogenesis, immune evasion and stem cell maintenance [2, 3].

TGF-β binds and activates a membrane receptor serine/threonine kinase complex that phosphorylates various Smad family proteins [4, 5]. Phosphorylated Smad2 levels have been proposed as a negative prognostic marker in glioblastoma [6]. In some cell types, TGF-β also signals via the phosphorylation of Smad1 and Smad5 [7]. Upon phosphorylation, Smads accumulate in the nucleus, form transcriptional complexes with Smad4 and other transcription factors, and regulate transcription [5]. TGF-β induces the expression of genes regulating the cell cycle and extracellular matrix, including plasminogen activator inhibitor (PAI)-1 [8] and platelet-derived growth factor (PDGF)-B [6]. Of note, TGF-β also activates, in a Smad-independent manner, important effector pathways for tyrosine kinase receptors, including PKB/Akt and extracellular regulated kinase (ERK) [9, 10]. TGF-β is a strong inhibitor of proliferation in epithelial cells, astrocytes, and immune cells, and is considered to represent a tumor suppressor factor. However, some malignant tumors, including gliomas, acquire mutations in elements of the TGF-β pathway that allow escape from the antiproliferative effects of TGF-β [3, 11], thus facilitating the pro-tumorigenic activity of TGF-β. This activity includes autocrine control of intrinsic tumor cell behavior as well as the modulation of the microenvironment and host responses. The relative dominance of these activities may vary between and even within distinct tumor entities. A better understanding of how to identify a cancer that critically depends on TGF-β signaling would greatly aid the development of targeted interventions.

## RESULTS

### Patient characteristics

Table 1 summarizes the principle patient characteristics. The median age of newly diagnosed glioblastoma patients (n=64) was 58 years, the majority having received TMZ/RT→TMZ (50%) as initial therapy, and the median survival was 16 months. A second group of 15 patients with progressive glioblastoma eligible for second and in one case for third surgery was also studied. The median age at diagnosis (57) was similar to the group of newly diagnosed glioblastoma, but the median overall survival was 26 months, representing a selected patient population. Individual patient profiles are summarized in Supplementary Table 2.

**Table 1:** Patient characteristics

	Newly diagnosed glioblastoma (n=64)	Recurrent glioblastoma (n=15+1)**
*Age (years)*		
*at diagnosis*		
Median	58	57
Range	1-85	18-72
*at recurrence*		
Median	n.a.	58
Range	n.a.	19-74
Age classes, n (%)*		
≤ 50 years	17 (26.6)	6 (37.5)
51-60 years	20 (31.3)	6 (37.5)
61-70 years	16 (25)	3 (18.8)
> 70 years	11 (17.2)	1 (6.3)
*Gender*		
Female	31 (48.4)	5 (31.3)
Male	33 (51.6)	11 (68.8)
KPS (pre-operative), n (%)*		
90 – 100	19 (29.7)	7 (43.8)
70 – 80	31 (48.4)	8 (50)
< 70	12 (18.8)	0 (0)
No data	2 (3.1)	1 (6.3)
*Tumor location at diagnosis*		
Frontal	17 (26.6)	3 (18.8)
Parietal	6 (9.4)	3 (18.8)
Temporal	19 (29.7)	5 (31.3)
Occipital	2 (3.1)	1 (6.3)
Not localized to one side	7 (10.9)	2 (12.5)
Multifocal	12 (18.8)	1 (6.3)
No data	1 (1.6)	1 (6.3)
Surgery, n (%)*		
Gross total resection	20 (31.3)	5 (31.3)
Incomplete resection	43 (67.2)	10 (62.5)
Biopsy	0 (0)	0 (0)
No data	1 (1.6)	1 (6.3)
*Histological subtype, n (%)*		
Glioblastoma	57 (89)	13 (81.3)
Glioblastoma with oligodendroglial component	6 (9.4)	3 (18.8)
Giant cell glioblastoma	1 (1.6)	0 (0)
*MGMT promoter methylation status, n (%)*		
Unmethylated	20 (31.3)	5 (31.3)
Weakly methylated	3 (4.7)	0 (0)
Methylated	5 (7.8)	1 (6.3)
No data	36 (56.3)	10 (62.5)
*First line therapy, n (%)*		
RT alone	11 (17.2)	5 (31.3)
CT alone	5^⌘^ (7.8)	0 (0)
RT plus TMZ	40 (62.5)	10 (62.5)
No therapy	4 (6.3)	0 (0)
No data	4 (6.3)	1 (6.3)
*Survival*		
*from diagnosis*		
Median follow-up (months)	13	22
Median PFS (months) (95% CI) (events)	5 (4.1-6.4) (54)	9 (5.3-12.0)
Median OS (months) (95% CI) (events)	16 (13.0-22.2) (40)	26 (17.3-31.1) (15)
Alive at last follow up (%)	37.5	25
*from recurrence*		
Median follow-up (months)	n.a.	11
Median PFS (months) (95% CI) (events)	n.a.	3 (0-11.1) (15)
Median OS (months) (95% CI) (events)	n.a.	13 (5.2-16.2) (16)

n.a., not applicable

* data are reported from the date of surgery tissue was obtained

** recurrent tumor tissues of two different surgeries were obtained from one patient

⌘ nitrosourea plus bevacizumab (n=1), temozolomide (n=4); PFS, progression-free survival; OS, overall survival; CI, confidence interval; RT, radiotherapy; CT, chemotherapy; MGMT, O^6^-methylguanyl-DNA-methyltransferase.

### Expression of TGF-β mRNA isoforms in glioblastoma *in vivo*

**Figure 1:**
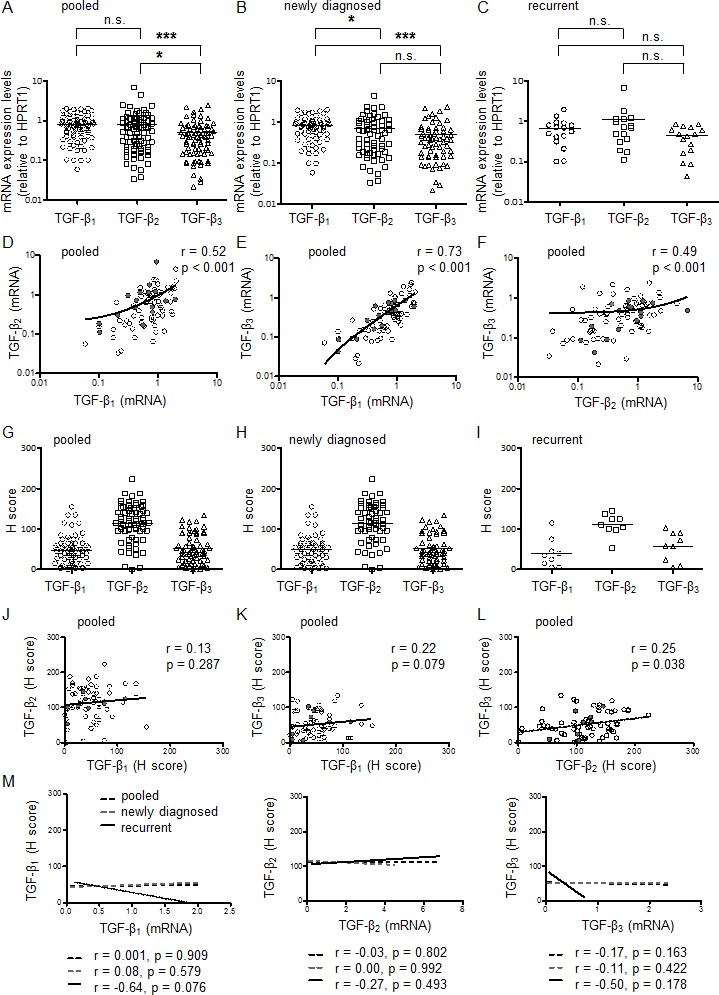
Expression of TGF-β isoforms in glioblastoma *in vivo* A-C, Relative mRNA expression levels for TGF-β_1_, TGF-β_2_ and TGF-β_3_ were assessed in all glioblastoma tissues (pooled, newly diagnosed or recurrent). The black bar marks the mean in each group. Values are represented on a logarithmic scale. Statistical significances of p < 0.05 (*) and p < 0.001 (***) were determined using the Mann-Whitney test. D-F, Correlation of TGF-β isoform mRNA expression among all samples pooled. Values are represented on a logarithmic scale. Two-tailed Spearman test coefficients (r) and significances are indicated (open circles, newly diagnosed; closed circles, recurrent). TGF-β_1_, TGF-β_2_ or TGF-β_3_ protein levels were assessed by immunohistochemistry and median H scores determined and presented for all patients pooled (G), newly diagnosed tumor tissues (H) and recurrent tumor tissues (I) separately. The black bar marks the mean in each group. Statistical significances of p < 0.01 (**) and p < 0.001 (***) were determined using the Mann-Whitney test. (J-L) Correlation of TGF-β protein levels among all samples pooled (open circles, newly diagnosed; closed circles, recurrent). (M) Correlation analyses of the three TGF-β protein levels with mRNA expression of the respective TGF-β isoform are shown. Two-tailed Spearman test coefficients (r) and significances are indicated.

TGF-β_1,2,3_ gene expression was measured by RT-PCR in 79 surgical specimens from 74 patients, including 5 patients with both primary and recurrent tumor specimens and 1 patient with recurrent tumor tissue from two different surgeries. All three TGF-β isoforms were expressed heterogeneously in glioblastoma samples (Supplementary Figure 1A-C). The median relative expression levels were 0.75 for TGF-β_1_ (95% CI 0.68 – 0.90), 0.54 for TGF-β_2_ (95% CI 0.58 – 1.03) and 0.39 for TGF-β_3_ (95% CI 0.39 – 0.61). TGF-β_1_ (p<0.001) and TGF-β_2_ (p=0.028) were more abundant than TGF-β_3_ mRNA for all patients pooled, whereas the expression levels between TGF-β_1_ and TGF-β_2_ did not differ significantly (Figure 1A). Median relative expression levels were 0.79 for TGF-β_1_ (95% CI 0.7 – 0.95), 0.52 for TGF-β_2_ (95% CI 0.54 – 0.93) and 0.38 for TGF-β_3_ (95% CI 0.38 – 0.65) in newly diagnosed tumors, and 0.63 for TGF-β_1_ (95% CI 0.42 – 0.93), 0.71 for TGF-β_2_ (95% CI 0.23 – 1.93) and 0.46 for TGF-β_3_ (95% CI 0.29 – 0.58) in recurrent tumors. In the subgroup of newly diagnosed glioblastomas, TGF-β_1_ was expressed at higher levels than TGF-β_2_ (p=0.04) or TGF-β_3_ (p<0.001), whereas expression levels between TGF-β_2_ and TGF-β_3_ did not differ (Figure 1B). There were no significant changes in relative mRNA expression levels of the three TGF-β isoforms in the smaller group of recurrent tumor tissue samples (Figure 1C). A correlation was observed between TGF-β_1_ and either TGF-β_2_ (r=0.52, p<0.001) (Figure 1D) or TGF-β_3_ mRNA (r=0.73, p<0.001) (Figure 1E), and between TGF-β_2_ and TGF-β_3_ mRNA levels (r=0.49, p<0.001) (Figure 1F) for all patients pooled (Table 2). When newly diagnosed and progressive tumors were analyzed separately (Supplementary Table 3), a correlation was noted between TGF-β_1_ and either TGF-β_2_ (newly diagnosed r=0.55, p<0.001; recurrent r=0.52, p=0.039) (Supplementary Figure 2A, D) or TGF-β_3_ mRNA (newly diagnosed r=0.74, p<0.001; recurrent r=0.83, p<0.001) (Supplementary Figure 2B, E) and also between TGF-β_2_ and TGF-β_3_ (newly diagnosed r=0.46 p<0.001; recurrent r=0.59, p=0.015) (Supplementary Figure 2C, F) mRNA.

**Table 2:** Correlation analyses for samples from newly diagnosed and progressive glioblastoma (pooled)

	TGF-β_2_ mRNA	TGF-β_3_ mRNA	TGF-β_1_ protein IHC	TGF-β_2_ protein IHC	TGF-β_3_ protein IHC	pSmad2 protein IHC	pSmad1/5/8 protein IHC	PDGF-B mRNA	PAI-1 mRNA	PAI-1 protein IHC	Ki-67 protein IHC
**TGF-β_1_ mRNA**	r = 0.52 p < 0.001***	r = 0.73 p < 0.001***	r = 0.01 p = 0.909 n.s.	r = 0.12 p = 0.334 n.s.	r =−0.1 p = 0.438 n.s.	r = −0.05 p = 0.693 n.s.	r = −0.06 p = 0.653 n.s.	r = 0.77 p < 0.001***	r = 0.55 p < 0.001***	r = 0.07 p = 0.608 n.s.	r = −0.16 p = 0.193 n.s.
**TGF-β_2_ mRNA**		r = 0.49 p < 0.001***	r = −0.04 p = 0.780 n.s.	r = −0.03 p = 0.802 n.s.	r = −0.07 p = 0.559 n.s.	r = −0.08 p = 0.489 n.s.	r = −0.11 p = 0.372 n.s.	r = 0.41 p < 0.001***	r = 0.36 p = 0.001**	r = 0.01 p = 0.939 n.s.	r = −0.02 p = 0.88 n.s.
**TGF-β_3_ mRNA**			r = −0.04 p = 0.765 n.s.	r = −0.04 p = 0.768 n.s.	r = −0.17 p = 0.163 n.s.	r = −0.08 p = 0.536 n.s.	r = −0.21 p = 0.087 n.s.	r = 0.69 p < 0.001***	r = 0.36 p < 0.001***	r = −0.16 p = 0.255 n.s.	r = 0.00 p = 0.98 n.s.
**TGF-β_1_ protein IHC**				r = 0.13 p = 0.287 n.s.	r = 0.22 p = 0.079 n.s.	r = 0.2 p = 0.114 n.s.	r = 0.51 p < 0.001***	r = −0.06 p = 0.62 n.s.	r = 0.14 p = 0.273 n.s.	r = 0.25 p = 0.074 n.s.	r = −0.11 p = 0.404 n.s.
**TGF-β_2_ protein IHC**					r = 0.25 p = 0.038*	r = −0.01 p = 0.928 n.s.	r = 0.10 p = 0.431 n.s.	r = 0.14 p = 0.263 n.s.	r = 0.2 p = 0.113 n.s.	r = 0.13 p = 0.368 n.s.	r = −0.17 p = 0.189 n.s.
**TGF-β_3_ protein IHC**						r = 0.03 p = 0.787 n.s.	r = 0.18 p = 0.162 n.s.	r = 0.04 p = 0.772 n.s.	r = 0.18 p = 0.138 n.s.	r = 0.09 p = 0.549 n.s.	r = −0.15 p = 0.233 n.s.
**pSmad2 protein IHC**							r = 0.24 p = 0.048*	r = 0.09 p = 0.48 n.s.	r = −0.10 p = 0.41 n.s.	r = 0.2 p = 0.159 n.s.	r = 0.15 p = 0.213 n.s.
**pSmad1/5/8 protein IHC**								r = −0.16 p = 0.206 n.s.	r = −0.01 p = 0.953 n.s.	r = 0.16 p = 0.273 n.s.	r = − 0.27 p = 0.029*
**PDGF-BmRNA**									r = 0.34 p = 0.002**	r = −0.22 p = 0.129 n.s.	r = −0.19 p = 0.135 n.s.
**PAI-1mRNA**										r = 0.29 p = 0.038*	r = −0.06 p = 0.645 n.s.
**PAI-1 protein IHC**											r = −0.07 p = 0.658 n.s.

Statistical significances of p < 0.05 (*), p < 0.01 (**) and p < 0.001 (***) were determined using the two-tailed Spearman correlation test (r, correlation coefficient). mRNA, messenger ribonucleic acid, IHC, immunohistochemistry, n.s., not significant.

No regulation of mRNA expression levels of either TGF-β isoform was found when newly diagnosed and recurrent glioblastomas were compared: TGF-β_1_ (p=0.30), TGF-β_2_ (p=0.21), TGF-β_3_ (p=0.56). Five paired samples of patients undergoing second surgery showed no consistent change, although there were striking parallel changes among the three TGF-β isoforms in the individual patients (Supplementary Figure 3A-C). Two out of the five paired samples (patients A,D) showed increased mRNA expression levels of TGF-β_1-3_ after recurrence, and 3 patients (patients B,C,E) showed decreased mRNA expression levels of TGF-β_1-3_ after recurrence.

### Expression of TGF-β proteins in glioblastoma *in vivo*

**Figure 2:**
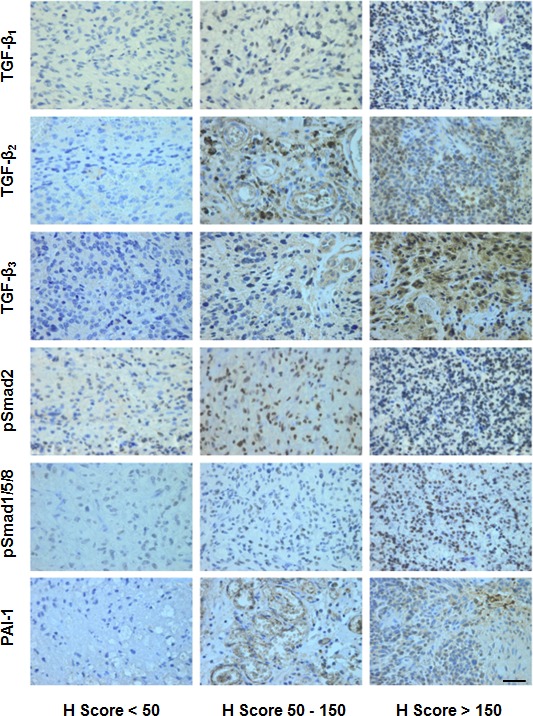
Immunohistochemical studies of the TGF-β pathway in glioblastoma Representative stainings for TGF-β_1_, TGF-β_2_, TGF-β_3_, pSmad2, pSmad1/5/8, and PAI-1 (score < 50 left, score 50-150 middle, score > 150 right). Size bars correspond to 100 μm.

Next, protein levels of all three TGF-β isoforms were assessed by IHC in 67 tissue samples, comprising 58 newly diagnosed and 9 recurrent tumor tissues. TGF-β_1-3_ protein levels varied considerably among glioblastoma samples *in vivo* (Figure 2, Supplementary Figure 1). The median H scores were 45 for TGF-β_1_ (95% CI 40 – 57), 116 for TGF-β_2_ (95% CI 102 – 125) and 46 for TGF-β_3_ (95% CI 43 – 60) for all patients pooled (Figure 1G), 48 for TGF-β_1_ (95% CI 40 – 59), 118 for TGF-β_2_ (95% CI 101 – 127) and 45 for TGF-β_3_ (95% CI 42 – 60) for newly diagnosed (Figure 1H) and 35 for TGF-β_1_ (95% CI 13 – 68), 111 for TGF-β_2_ (95% CI 90 – 132) and 59 for TGF-β_3_ (95% CI 29 – 85) for recurrent glioblastomas (Figure 1I). There was significant correlation between TGF-β_2_ and TGF-β_3_ (r=0.25, p=0.038) (Figure 1L), but not between TGF-β_1_ and TGF-β_2_ (r=0.13, p=0.287) (Figure 1J) or between TGF-β_1_ and TGF-β_3_ (r=0.22, p=0.079) (Figure 1K) protein levels for all patients pooled (Table 2). Separate correlation analyses between protein levels of the TGF-β did not reach significance in the subgroups of newly diagnosed (Supplementary Figure 2G-I) or recurrent tumors (Supplementary Figure 2J-L, Supplementary Table 3).

Interestingly, protein levels of the three TGF-β did not show significant correlation with mRNA expression levels of the respective TGF-β isoforms (TGF-β_1_ r=0.01, p=0.909, TGF-β_2_ r=−0.03, p=0.802, TGF-β_3_ r=−0.17, p=0.163) for all patients pooled (Figure 1M, Table 2). Similar results were observed when newly diagnosed and progressive tumors were analyzed separately (Figure 1M, Supplementary Table 3).

### Assessment of TGF-β pathway activation in glioblastoma: Smad phosphorylation

**Figure 3:**
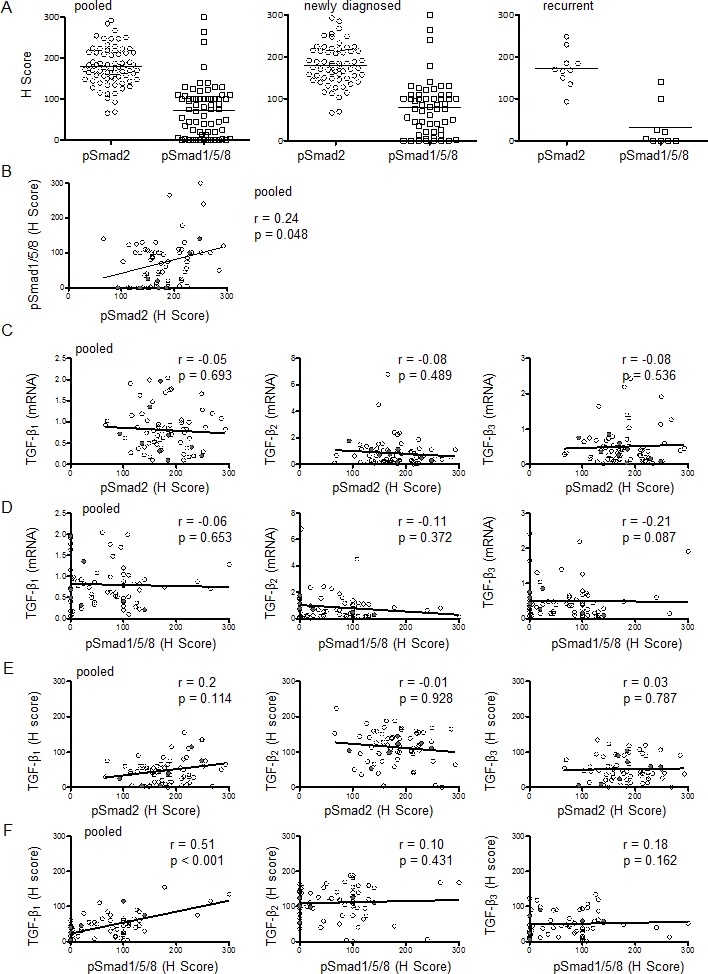
Assessment of TGF-β pathway activation in glioblastoma: Smad phosphorylation A, pSmad2 or pSmad1/5/8 protein levels were assessed by immunohistochemistry and median H Scores are shown for all patients pooled, newly diagnosed tumor tissues or recurrent tumor tissues separately. The black bar marks the mean in each group. B, Correlation is shown for the H scores of pSmad2 and pSmad1/5/8 for all samples pooled. Paired correlation analyses of pSmad2 (C,E) or pSmad1/5/8 (D,F) protein levels and TGF-β isoform mRNA (C,D) or protein (E,F) levels are shown for all samples pooled. Two-tailed Spearman test coefficients (r) and significances (p) are indicated (open circles, newly diagnosed; closed circles, recurrent).

To analyze the activity of the canonical TGF-β pathway in glioblastoma, we determined the levels of pSmad2 and pSmad1/5/8 as the main targets of TGF-β superfamily-dependent signal transduction. Immunohistochemical analysis showed that pSmad2 and pSmad1/5/8 were mainly localized in the nucleus and that the intensity of the staining varied between tumor samples (Figure 2, Supplementary Figure 1). The median H Score (range 0 – 300) was 175 (95% CI 168 – 191) for pSMAD2 and 75 (95% CI 57 – 88) for pSmad1/5/8 for all patients pooled (Figure 3A). In the subgroups of newly diagnosed or recurrent patients, the median H Scores were 178 (95% CI 168 – 193) or 169 (95% CI 141 – 204) for pSMAD2 and 79 (95% CI 62 – 96) or 3 (95% CI −7.80 – 72) for pSMAD1/5/8. There was a positive correlation between pSmad2 and pSmad1/5/8 protein levels (r=0.24, p=0.048) (Figure 3B, Table 2) that did not reach significance in the subgroups of newly diagnosed or recurrent tumors in separate analyses (Supplementary Table 3). Correlation analyses were performed between mRNA data of the three TGF-β isoforms and protein data of pSmad2 (Figure 3C) or pSmad1/5/8 (Figure 3D), demonstrating no significance. These results are in line with data assessed in the subgroup of newly diagnosed patients (Supplementary Figure 4A,B), whereas there was a negative correlation between pSmad2 and mRNA expression levels of TGF-β_2_ (r=−0.67, p=0.039) or TGF-β_3_ (r=−0.67, p=0.039) and between pSmad1/5/8 and mRNA expression levels of TGF-β_2_ (r=−0.72, p=0.031) in the small subgroup of recurrent tumor patients (Supplementary Figure 4C, D).

Moreover, no significant correlation was observed between TGF-β_1_ protein (r=0.2, p=0.114), TGF-β_2_ protein (r=−0.01, p=0.928) or TGF-β_3_ protein (r=0.03, p=0.787) levels and pSmad2 protein levels, when all patients were analyzed together (pooled) (Figure 3E, Table 2) or per subgroup (Supplementary Figure 4E, G, Supplementary Table 3). There was significant correlation between protein levels of TGF-β_1_ and protein levels of pSmad1/5/8 (r=0.51 p<0.001) for all patients pooled (Figure 3F, Table 2), as well as in the glioblastoma subgroups of newly diagnosed and recurrent tumors (Supplementary Figure 4 F,H). Correlation analyses of protein levels of TGF-β_2_ (r=0.10, p=0.431) or TGF-β_3_ (r=0.18, p=0.162) and pSmad1/5/8 protein levels did not reach significance for all patients pooled (Figure 3F, Table 2) and in the subgroup analyses (Supplementary Figure 4F,H, Supplementary Table 3).

### Assessment of TGF-β pathway activation in glioblastoma: expression of TGF-β response genes

**Figure 4:**
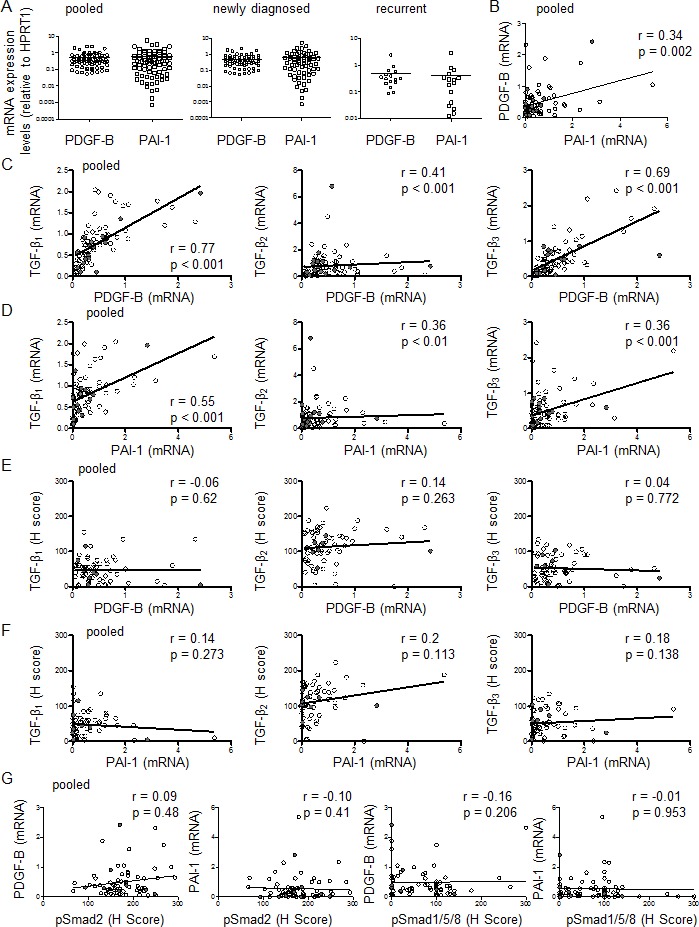
Assessment of TGF-β pathway activation in glioblastoma: expression of TGF-β response genes A, PDGF-B or PAI-1 mRNA expression data were assessed by RT-PCR for all patients pooled, newly diagnosed tumor tissues or recurrent tumor tissues separately. The black bar marks the mean in each group. Values are represented on a logarithmic scale. B, Correlation is shown for the mRNA data of PDGF-B and PAI-1 for all samples pooled. Correlation was assessed of mRNA data of TGF-β_1_ or TGF-β_2_ or TGF-β_3_ and mRNA data of PDGF-B (C) or PAI-1 (D) for all patients pooled. Correlation was assessed of protein data of TGF-β_1_ or TGF-β_2_ or TGF-β_3_ and mRNA data of PDGF-B (E) or PAI-1 (F) for all patients pooled. G, Correlation is shown for pSmad2 or pSmad1/5/8 mRNA data and PDGF-B or PAI-1 mRNA data. Two-tailed Spearman test coefficients (r) and significances (p) are indicated (open circles, newly diagnosed; closed circles, recurrent tumor specimens).

The mRNA expression of PDGF-B and PAI-1, two bona fide response genes of TGF-β, was also assessed (Supplementary Figure 1). The median relative expression levels were 0.34 (95% CI 0.38–0.59) for PDGF-B and 0.29 (95% CI 0.38–0.75) for PAI-1 for all patients pooled, 0.36 (95% CI 0.37–0.60) for PDGF-B and 0.30 (95% CI 0.38–0.83) for PAI-1 in newly diagnosed patients, and 0.32 (95% CI 0.18–0.78) for PDGF-B and 0.25 (95% CI 0.05–0.78) for PAI-1 in recurrent tumors (Figure 4A). Median expression levels for PDGF-B or PAI-1 did not differ significantly among the three groups. In fact, their relative expression levels correlated well (r=0.34, p=0.002) for all patients pooled (Figure 4B, Table 2). When newly diagnosed and progressive tumors were analyzed separately, there was still significant correlation between PDGF-B and PAI-1 in newly diagnosed tumors (r=0.32, p=0.009), but not in the subgroup of recurrent tumors (r=0.37, p=0.158) (Supplementary Table 3).

Next we compared TGF-β mRNA expression with TGF-β target gene expression. There was correlation between PDGF-B and TGF-β_1_ (r=0.77, p<0.001), TGF-β_2_ (r=0.41, p<0.001) or TGF-β_3_ mRNA (r=0.69, p<0.001) (Figure 4C), and similarly, between PAI-1 and TGF-β_1_ (r=0.55, p<0.001), TGF-β_2_ (r=0.36, p=0.001) or TGF-β_3_ mRNA (r=0.36, p<0.001) (Figure 4D) for all patients pooled (Table 2). In the subgroup of newly diagnosed tumors, there was correlation between mRNA data of PDGF-B and TGF-β_1_ (r=0.78, p<0.001), TGF-β_2_ (r=0.42, p<0.001) or TGF-β_3_ (r=0.73, p<0.001), and between PAI-1 and TGF-β_1_ (r=0.54, p<0.001), TGF-β_2_ (r=0.39, p=0.001) or TGF-β_3_ (r=0.36, p=0.004) (Supplementary Figure 5A, B, Supplementary Table 3). In the subgroup of recurrent glioblastomas, significant correlation of mRNA data was found between TGF-β_1_ and PDGF-B (r=0.62, p=0.011) or PAI-1 (r=0.54, p=0.033), but not between TGF-β_2_ and PDGF-B (r=0.32, p=0.226) or PAI-1 (r=0.31, p=0.226) or TGF-β_3_ and PDGF-B (r=0.46, p=0.072) or PAI-1 (r=0.32, p=0.226) (Supplementary Figure 5C, D, Supplementary Table 3). mRNA expression levels for PDGF-B and PAI-1 were also analyzed separately for the five paired samples of patients undergoing second surgery. No consistent change was demonstrated (Supplementary Figure 3F), although parallel changes between PDGF-B and PAI-1, also in comparison to the three TGF-β isoforms in the individual patients, were observed (Supplementary Figure 3D,E).

In contrast, TGF-β protein levels of all three TGF-β isoforms did not correlate with the mRNA data of PDGF-B (TGF-β_1_: r=−0.06, p=0.62, TGF-β_2_: r=0.14, p=0.263 or TGF-β_3_: r=0.04, p=0.772) (Figure 4E, Table 2) or PAI-1 (TGF-β_1_: r=0.14, p=0.273, TGF-β_2_: r=0.2, p=0.113 or TGF-β_3_: r=0.18, p=0.138) (Figure 4F, Table 2). In the subgroups of newly diagnosed and recurrent tumors, no significant correlation between TGF-β protein isoforms and mRNA data for PDGF-B or PAI-1 was observed either (Supplementary Figure 5E-H, Supplementary Table 3). Further, pSmad2 or pSmad1/5/8 protein levels did not correlate with mRNA expression levels of PDGF-B (r=0.09, p=0.48 or r=−0.16, p=0.206) or PAI-1 (r=−0.10, p=0.41 or r=−0.01, p=0.953), either for all patients pooled (Figure 4G, Table 2) or for the subgroups (Supplementary Table 3). We also determined PAI-1 protein levels by immunohistochemistry (Figure 2, Supplementary Figure 1). The median H score for PAI-1 was very low: 4 (95% CI 7-20) for all patients pooled, 5 (95% CI 7-22) for newly diagnosed and 3 (95% CI 0-16) for recurrent tumors (Supplementary Figure 6A). PAI-1 protein data correlated with PAI-1 mRNA data (r=0.29, p=0.038), but not with PDGF-B mRNA data (r=−0.22, p=0.129) for all patients pooled (Supplementary Figure 6B). No significant correlation was observed between PAI-1 protein levels and levels of TGF-β_1_ mRNA (r=0.07, p=0.608), TGF-β_2_ mRNA (r=0.01, p=0.939) or TGF-β_3_ mRNA (r=−0.16, p=0.787) (Supplementary Figure 6C, Table 2), or levels of TGF-β_1_ protein (r=0.25, p=0.074), TGF-β_2_ protein (r=0.13, p=0.368) or TGF-β_3_ protein (r=0.09, p=0.549) (Supplementary Figure 6D, Table 2), when all patients were analyzed together (pooled). Correlation analyses between protein levels of PAI-1 and pSmad2 (r=0.20, p=0.159) or pSmad1/5/8 (r=0.16, p=0.273) also failed to reach significance (Supplementary Figure 6E, Table 2). Similar results were observed in the glioblastoma subgroups (Supplementary Table 3).

### TGF-β expression and proliferation

Ki-67 protein labeling as a surrogate marker of proliferation was not significantly associated with the TGF-β isoform mRNA or protein level, neither for all patients pooled (Table 2) nor for subgroups of patients analyzed for newly diagnosed or recurrent tumors (Supplementary Table 3). Similarly, Ki-67 labeling did not correlate with pSmad2 levels for all patients pooled (r=0.15, p=0.21) (Table 2, Supplementary Figure 7A), or for the subgroups of patients with newly diagnosed or recurrent tumors (Supplementary Figure 7B,C, Supplementary Table 3). In contrast, Ki-67 correlated inversely with pSmad1/5/8 levels for all patients pooled (r=−0.27, p=0.029) (Table 2, Supplementary Figure 7A) and more so in newly diagnosed tumors (r=−0.35, p=0.008), although not in the small group of recurrent tumors (r=−0.24, p=0.58) (Supplementary Figure 7B,C, Supplementary Table 3). Finally, Ki-67 protein labeling was not associated with PDGF-B or PAI-1 mRNA expression or PAI-1 protein levels, neither for all patients pooled (Table 2) nor for the subgroups of patients with newly diagnosed versus recurrent tumors (Supplementary Table 3).

### TGF-β pathway activity and age

Correlation analyses were also performed to identify whether patient age played a role for TGF-β dependency in newly diagnosed glioblastomas. Only one patient included in this study was under 18 years old and was censored for this analysis. Moreover, only patients with newly diagnosed glioblastoma were included. Age at diagnosis correlated well with TGF-β_2_ mRNA expression levels (r=0.33, p=0.009), TGF-β_3_ mRNA expression levels (r=0.26, p=0.045) and PAI-1 mRNA expression levels (r=0.33, p=0.009). In contrast, no significant correlation was found for age and TGF-β_1_ mRNA expression levels, TGF-β protein levels, PDGF-B mRNA expression levels, Ki-67, pSmad2, pSmad1/5/8 or PAI-1 protein levels (Supplementary Table 4).

### TGF-β pathway activity and outcome

To assess for an association between TGF-β and survival in the group of newly diagnosed tumors, tumors were divided into “high” and “low” groups, with “high” defined as higher than the median and “low” as lower or equal to the median. Survival estimated by the Kaplan-Meier method was then compared with the two-sided log-rank test. Expression levels of mRNA or protein of targets of the TGF-β/Smad pathway activity did not reveal an association with survival, when the median expression level was used as the cut-off (Supplementary Figure 8A). As a sensitivity analysis, these outcome studies were also performed for all patients who received at least RT, thus omitting P13, P16, P17, P18, P23, P29, P32, P35, P39, P45, P56, P60 and P62, but no prognostic role for any parameter studied was confirmed (data not shown). When the expression cut-off was placed by Graphpad to yield the highest correlation with outcome, a very small group of patients with high expression levels of TGF-β_2_ (mRNA) (p=0.048) or pSmad1/5/8 (protein) (p=0.032) showed a reduced probability of survival (Supplementary Figure 8B).

### TGF-β pathway activity and outcome: an analysis of the Cancer Genome Atlas (TCGA) network

**Figure 5:**
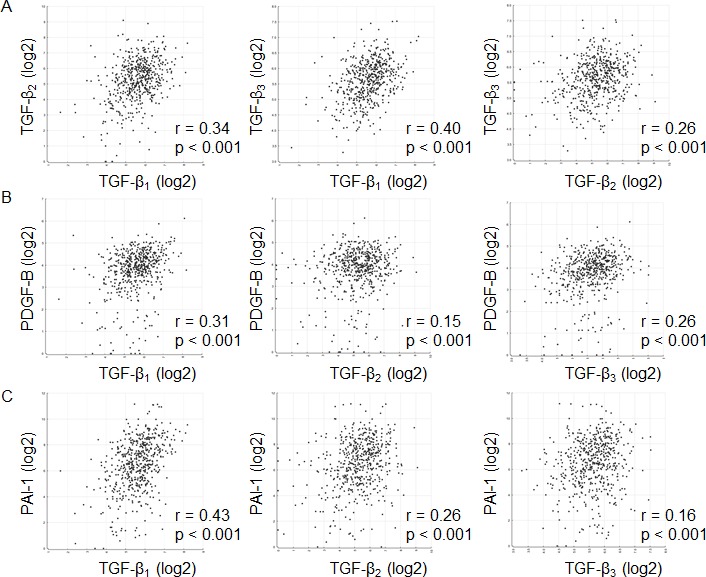
TGF-β pathway activity: an analysis of the Cancer Genome Atlas (TCGA) network A, Correlation is shown among the TGF-β isoforms. Correlation was assessed using mRNA data of TGF-β_1_ or TGF-β_2_ or TGF-β_3_ and mRNA data of PDGF-B (B) or PAI-1 (C). Two-tailed Spearman test coefficients (r) and significances (p) are indicated. Data are obtained from the TCGA network.

Microarray and clinical data for TGF-β_1-3_, PDGF-B or PAI-1 mRNA in glioblastoma patients were acquired from the TCGA database [12] (Supplementary Table 1). Correlation analyses were assessed between TGF-β isoforms (Figure 5A) and between TGF-β isoforms and PDGF-B (Figure 5B) or PAI-1 (Figure 5C). These analyses demonstrated a strong positive correlation for all data tested. We also asked whether any of these genes were differentially expressed in the molecular subtypes classified by Verhaak et al. [13]. mRNA data of different expression subtypes were available from 473 glioblastoma patients (n=96, proneural; n=83, neural; n=152, mesenchymal; n=142, classical) (Supplementary Figure 9A). TGF-β_1_ mRNA levels were significantly higher in mesenchymal glioblastoma than in proneural (p<0.001), neural (p<0.001) or classical glioblastoma (p<0.001). TGF-β_2_ mRNA levels were increased in mesenchymal and classical glioblastoma, compared with the two other subgroups (proneural p<0.001 and p<0.001, neural p=0.008 and p<0.001), whereas expression levels did not differ between mesenchymal and classical glioblastoma (p=0.515). Similar results were observed for TGF-β_3_ mRNA expression levels. PDGF-B was increased in the classical subtype, compared with the proneural (p=0.007) and neural (p=0.022) subtype. Highest mRNA expression levels of PAI-1 were found in the mesenchymal subtype compared to all other expression subtypes (proneural p<0.001, neural p<0.001 and classical p<0.001). Sequencing data were available from 229 of the 473 glioblastoma patients. 8 of 78 mesenchymal glioblastomas, but only 4 of 151 non-mesenchymal glioblastomas had mutations in the neurofibromatosis (NF) 1 gene. TGF-β_3_ mRNA levels were higher in patients with tumors with mutations in the NF1 gene, for all patients pooled (p=0.013) and also in patients diagnosed for the mesenchymal glioblastoma subgroup (p=0.03) (Supplementary Figure 9B). Neither TGF-β_1_ or TGF-β_2_ gene nor TGF-β target gene expression differed by NF1 mutation status, irrespective of whether all glioblastomas pooled or only mesenchymal tumors were analyzed, with the exception of PAI-1, which was increased in patients with NF1 mutations for all patients pooled (p=0.048), but not for the subgroup of mesenchymal tumors (p=0.663) (Supplementary Figure 9B).

The survival analysis of glioblastoma patients of the TCGA database revealed no association with survival for the five targets (TGF-β_1-3_, PDGF-B or PAI mRNA) when the median expression level defined the cut-off for dividing glioblastoma patients into those with high or low expression (Supplementary Figure 10A). However, enhanced expression of TGF-β_1-3_, PDGF-B or PAI mRNA was associated with inferior survival when the expression cut-off was defined individually for the statistically ideal cut-off (Supplementary Figure 10B).

## DISCUSSION

TGF-β has emerged as one of the most promising, but also challenging targets of cancer therapy [9]. Specifically in glioblastoma, various pharmacological approaches to limit TGF-β pathway activity have been explored, based on the putative role of TGF-β in migration, invasiveness, angiogenesis and immunosuppression. The most advanced approach was locoregional using TGF-β_2_ antisense oligonucleotides, which was investigated in a randomized phase II trial in patients with recurrent anaplastic gliomas or glioblastomas. This treatment appeared not to be inferior to alkylating agent chemotherapy, although the efficacy data of that trial remained essentially inconclusive [14, 15]. Further, a TGF-β type I receptor antagonist, LY2157299, is currently explored in clinical trials in recurrent as well as newly diagnosed glioblastoma [16, 17].

One major limitation of current approaches to exploit TGF-β as a target for therapy in glioblastoma as well as in other cancers is the lack of strategies to identify which tumors or patients are likely to respond to TGF-β inhibition. In part this is due to the fact that it remains controversial whether tumor or patient characteristics are more relevant in the context of TGF-β inhibition, that is, what are the relative contributions of autocrine or paracrine activities of TGF-β as opposed to TGF-β effects on the host in the pathogenesis of glioblastoma.

We find that all three TGF-β isoforms are broadly expressed in glioblastoma (Figure 1), suggesting that targeted approaches focusing on a particular isoform are unlikely to be successful in glioblastoma. Unexpectedly, the expression of all three isoforms of TGF-β seems to be controlled by a common pathway yet to be identified, given the striking correlation of TGF-β mRNA isoform expression. mRNA expression levels of the TGF-β isoforms were remarkably similar among newly diagnosed and recurrent tumors, indicating that the intercurrent treatment in this patient population with radiotherapy or radiochemotherapy does not durably down- or up-regulate TGF-β pathway activity (Supplementary Figures 1 and 3). The strong correlation between TGF-β mRNA expression and the mRNA expression of two bona fide target genes of TGF-β, PDGF-B and PAI-1, strongly suggests that glioblastomas are responsive to autocrine modulation by TGF-β. There was some indication of stronger TGF-β pathway activation with increasing patient age (Supplementary Table 4).

TGF-β gene and TGF-β target gene expression were increased in glioblastomas with mesenchymal gene expression signature (Supplementary Figure 9), which is defined by aberrations of the NF1 gene and proposed to be associated with inferior survival [13]. This may define a subgroup of patients more likely to benefit from TGF-β-targeted therapy.

We noted a weak correlation of TGF-β mRNA and protein levels, both at the level of relative quantities of TGF-β isoforms and at the levels of mRNA and protein of the same isoform. Technical issues of TGF-β assessment were optimized as feasible, but cannot be entirely excluded. Further explanations include differential posttranscriptional regulation among the TGF-β isoforms as well a non-glioma origin of some of the TGF-β molecules detected by immunohistochemistry.

That TGF-β target gene expression showed a stronger correlation with TGF-β mRNA expression than with Smad phosphorylation is difficult to interpret, assuming that TGF-β target gene expression is under the control of canonical TGF-β signaling. It may reflect the promiscousity of Smad complexes. The TGF-β superfamily includes more than 30 proteins, e.g., TGF-β, activins and inhibins, nodal, myostatin, BMP, growth differentiation factor (GDF) and anti-Müllerian hormone/Müllerian inhibiting substance (AMH/MIS). Five of the mammalian Smads - Smad1, Smad2, Smad3, Smad5, and Smad8 – act as substrates for the TGF-β receptor family and are designated as receptor-regulated Smads. Here, the pathway splits into two distinct branches down-stream of type I receptors, which are also known as activin receptor-like kinases (ALK). ALK4, ALK5 and ALK7 specifically phosphorylate Smad2 and Smad3, whereas ALK1, ALK2, ALK3 and ALK6 specifically phosphorylate Smad1, Smad5 and Smad8 [18]. Beyond that, possibly other pathways co-regulate pSmad2 levels in glioblastoma.

Finally, the absence of a strong correlation between any TGF-β-related parameter and Ki-67 labeling (Table 2) indicates that stimulation of proliferation is not an important role of the TGF-β pathway in glioblastoma. Yet, the inverse correlation of Ki-67 indexes with pSmad1/5/8 levels would be consistent with a negative control of glioblastoma growth by bone morphogenetic proteins (BMP), which also signal via pSmad1/5/8 [19]. Interestingly, this study provides evidence that pSmad1/5/8 may correlate with pSmad2 levels (Figure 3) and TGF-β_1_ protein levels (Figure 3) and that high pSmad1/5/8 protein levels are associated with inferior survival (Supplementary Figure 8 and 10). These observations call for further studies on the role of pSmad1/5/8 in glioblastoma, e.g., BMP antagonists may protect tumor cells from BMP-induced, pSmad1/5/8-mediated differentiation [20, 21].

With the identification of biomarkers for tumor or patient selection for future clinical trials targeting TGF-β as the major goal of this study, it is important to note that we had no opportunity to explore whether host cells, notably immune cells, were susceptible to tumor-derived TGF-β.

In summary, this study provides important new information on the biology of TGF-β in glioblastoma, in particular that (i) all three isoforms are expressed and biologically active, (ii) their expression seems to be commonly controlled, and (iii) determination of either TGF-β or TGF-β target gene mRNA expression may help to enrich for subgroups of glioblastomas characterized by TGF-β pathway activation.

## METHODS

### Patients

In accordance with the appropriate Institutional Review Boards, and following informed consent, the surgical specimens and clinical records were retrieved from 74 glioblastoma patients who underwent brain tumor resection between 12/2007 and 3/2012 at the Department of Neurosurgery, University Hospital Zurich, Zurich, Switzerland. Sixty-four newly diagnosed and 16 recurrent glioblastomas were studied. Both primary and recurrent tumor specimens were obtained from 5 patients, whereas recurrent tumor tissues from 2 different surgeries were studied from 1 patient. All tumors were classified and graded according to the WHO classification of tumors of the central nervous system [22]. Individual patient characteristics and O^6^-methylguanyl-DNA-methyltransferase (MGMT) status were taken from patient health records.

### Immunohistochemistry

Immunohistochemistry (IHC) was performed on formalin-fixed 4-μm-thick sections on SuperFrost slides (Menzel-Glaser, Braunschweig, Germany). Deparaffinized, rehydrated sections underwent heat-induced antigen retrieval by boiling in 10 mmol/l citrate buffer, pH 6.0 for 15 min in a steamer. Sections were treated with 1% H_2_O_2_ for 15 min to block endogenous peroxidase followed by a blocking step (tris-buffered saline (TBS) containing 10% swine serum, 0.2% Triton and 2% bovine serum albumin (BSA)) for 30 min at room temperature in a humid chamber. Immunostaining involved the sequential application of primary antibodies for Ki-67 (Dako, Cambridge, UK), pSmad2 (Cell Signaling Technology, Cambridge, UK), pSmad1/5/8 (Cell Signaling Technology), TGF-β_1_ (G1221, Promega, WI, USA), TGF-β_2_ (ab36495, Abcam) and TGF-β_3_ (AF-243-NA, R&D). The following secondary antibodies were used: for pSmad2 and pSmad 1/5/8 stainings biotinylated-SP-conjugated donkey anti-rabbit antibody (dilution 1:200) (JacksonImmuno, Newmarket, UK), for Ki67 immunohistochemistry, biotinylated rabbit anti-rat IgG antibody (dilution 1:200) (Burlingame, CA, USA), for TGF-β_1_ staining, Histofine Simple stain Max PO® Universal Immuno-Peroxidase Polymer anti-rabbit antibody (414141F, Nichirei Biosciences, Tokyo, Japan), for the TGF-β_2_ stainings biotinylated anti-mouse secondary antibody (dilution 1:200) (Vectastain ABC Kit PK-4002, Vector Laboratories, Peterborough, UK), and for TGF-β3 biotinylated rabbit anti-goat IgG antibody (1:200) (Vectastain ABC Kit PK-4002). Controls included the corresponding pre-immune antiserum or isotype-matched primary monoclonal antibody. In order to test the specificity of the TGF-β antibodies, immunoblotting with recombinant human TGF-β_1_, TGF-β_2_ or TGF-β_3_ was performed, ruling out cross-reactivity of the respective antibodies. For visualization, 3,3-diaminobenzidine tetrahydrochloride (Dako, Glostrup, Denmark) was used. Cytoplasmic staining was required to score a tumor cell as positive for TGF-β_1-3_, and nuclear staining for Ki-67, p-Smad2, and pSmad1/5/8. For the quantitative analysis of TGF-β_1-3_, p-Smad2, and p-Smad1/5/8, the percentage of stained tumor cells and intensity of staining were evaluated in representative high-power fields on tissue sections using light microscopy. The immunostaining results were expressed as H scores ranging from 0 – 300 and calculated as the percentage of weakly stained cells plus the percentage of moderately stained cells multiplied by two plus the percentage of strongly stained cells multiplied by three. For Ki-67, the percentage of stained tumor cell nuclei was calculated based on five elected high power fields with highest expression (40x magnification). Scoring was performed by J.S., L.E. and K.F. and supervised by E.R., all blinded to clinical data.

### Real-time PCR (RT-PCR)

Shock frozen tumor tissue (10-20 mg) was homogenized by a SilentCrusher S (Heidolph Instruments, Solothurn, Switzerland) in RA1 lysis buffer (Macherey-Nagel, Düren, Germany) containing 20 mM Tris (2-carboxyethyl)phosphine (TCEP). Total RNA was prepared using the NucleoSpin RNA II system (Macherey-Nagel) and cDNA transcribed using Superscript II reverse transcriptase (Invitrogen, Paisley, UK). For real-time PCR, cDNA amplification was monitored using SYBRGreen chemistry on the 7300 Real time PCR System (Applied Biosystems, Zug, Switzerland). The conditions for the PCR reactions were as follows: 40 cycles, 95°C/15 sec, 60°C/1 min, using the following specific primers:

PAI-1fwd 5′-CAGAAAGTGAAGATCGAGGTGA AC-3′,
PAI-1 rv 5′-GGAAGGGTCTGTCCATGATGAA-3′,
PDGF-B fwd: 5′-GAAGGGTCTGTCCA-3′,
PDGF-B rv: 5′-TCCAACTCGGCCCCATCT-3′,
TGF-β_1_ fwd: 5′-GCCCTGGACACCAACTATT G-3′,
TGF-β_1_ rv: 5′-CGTGTCCAGGCTCCAAATG-3′,
TGF-β_2_ fwd: 5′-AAGCTTACACTGTCCCTGCTG C-3′,
TGF-β_2_ rv: 5′-TGTGGAGGTGCCATCAATACC T-3′,
TGF-β_3_ fwd: 5′-TCAGCCTCTCTCTGTCCACTT-3′,
TGF-β_3_ rv: 5′-CATCACCGTTGGCTCAGGG-3′,
HPRT1 fwd: 5′-TGA GGATTTGGAAAGGGTGT-3′,
HPRT1 rv: 5′-GAGCACACAGAGGGCTACAA-3′.

HPRT1 transcript levels were used as house-keeping reference for relative quantification of mRNA expression levels using the ΔC_T_ method [23].

### Interrogations from The Cancer Genome Atlas (TCGA) network

Microarray and clinical data were obtained from the glioblastoma data set of the Cancer Genome Atlas network available on January 16, 2015. (http://cancergenome.nih.gov/) [12]. The gene expression data in this database were collected using Affymetrix gene chips. The query was based on the reporter with the highest mean geometric intensity for the target gene. The list of genes and Affymetrix probesets used in the TCGA database are summarized in Supplementary Table 1. Survival analyses within the glioblastoma data set of the TCGA database were performed using the Kaplan-Meier analysis module of the R2 microarray analysis and visualization platform (http://r2.amc.nl). Different cut-offs were defined to segregate glioblastoma patients into two groups with high or low expression of the target gene: specifically, cut-offs were defined by the median expression level and the highest association with survival. Sequencing data within the glioblastoma data set of the TCGA database were analyzed using the cBioPortal for Cancer Genomics (http://www.cbioportal.org) [24].

### Statistics

Progression-free survival (PFS) and overall survival (OS) curves were estimated by the Kaplan-Meier method and compared with the two-sided log-rank test. PFS was calculated from the time of surgery to the date of recurrence. Overall survival was measured from the date of surgical resection to the date of death. Patients without confirmed death were censored for overall survival at the last follow-up visit. Patients without documented progression were censored at the last follow-up visit for PFS and for overall survival. A Spearman correlation test was used to analyze relationships between individual parameters. The Mann-Whitney test was used to compare mRNA expression levels within groups (column statistics). Survival-related analyses were calculated with the log-rank test. All statistical analyses were performed using Prism 5 (GraphPad Software).

## SUPPLEMENTARY MATERIAL TABLES AND FIGURES


