# Supplementary material for: Transforming growth factor-β pathway activity in glioblastoma
Source: Oncotarget. 2015 Feb 28;6(8):5963–77. doi: 10.18632/oncotarget.3467 (PMC4467414; doi:10.18632/oncotarget.3467)
Supplement: Supplementary file 1 [file oncotarget-06-5963-s001.pdf]

Transforming growth factor-β pathway activity in glioblastoma

Supplementary Material

SUPPLEMENTARY TABLES

Supplementary Table 1: *List of genes and Affymetrix probesets used in the TCGA database.*

Affymetrix probeset ID	Gene symbol
203085_s_at	TGF-β_1
209909_s_at	TGF-β_2
209747_at	TGF-β_3
204200_s_at	PDGFB
202627_at	Serpine1*

*Serpine1 = PAI-1

Supplementary Table 2: *Individual patient characteristics.*

Patient	ID	Age	Age °	Gender	Disease status	Extent of Resection*	KPS (%)*	*MGMT* promotor methylation	First-line therapy	PD	PFS [months]	OS [months]
P1	ZH18	57	-	M	newly diagnosed	subtotal	80	n.d.	TMZ/RT→TMZ	yes	1	16
P2	ZH41	46	-	F	newly diagnosed	subtotal	90	n.d.	TMZ/RT→TMZ	yes	1	39
P3	ZH44	68	-	M	newly diagnosed	gross total	70	n.d.	TMZ/RT→TMZ	yes	12	24
P4_A	ZH45	57	-	F	newly diagnosed	gross total	70	n.d.	TMZ/RT→TMZ	yes	6	10
P5	ZH52	57	-	M	newly diagnosed	partial	60	n.d.	TMZ/RT→TMZ	yes	7	13
P6_B	ZH58	72	-	F	newly diagnosed	gross total	70	n.d.	RT	yes	12	29
P7	ZH79	50	-	M	newly diagnosed	subtotal	100	n.d.	TMZ/RT→TMZ	n.d.	60	60+
P8	ZH92	72	-	M	newly diagnosed	subtotal	80	n.d.	RT	yes	2	2+
P9	ZH104	65	-	M	newly diagnosed	subtotal	90	n.d.	TMZ/RT→TMZ	yes	33	48
P10	ZH109	51	-	M	newly diagnosed	subtotal	60	no	TMZ/RT→TMZ	yes	6	11+
P11	ZH120	47	-	M	newly diagnosed	subtotal	60	n.d.	RT -> TMZ	yes	1	1
P12	ZH121	40	-	M	newly diagnosed	partial	80	n.d.	TMZ/RT→TMZ	yes	9	10
P13	ZH125	56	-	M	newly diagnosed	subtotal	90	yes	n.d.	n.d.	0	0+
P14	ZH129	65	-	M	newly diagnosed	partial	70	yes	RT	yes	3	12
P15	ZH130	62	-	M	newly diagnosed	subtotal	70	no	TMZ/RT→TMZ	yes	2	6
P16	ZH131	51	-	F	newly diagnosed	gross total	100	n.d.	TMZ	yes	11	16+
P17	ZH133	1	-	M	newly diagnosed	gross total	n.d.	n.d.	TMZ	n.d.	47	47+
P18	ZH135	63	-	F	newly diagnosed	gross total	80	no	n.d.	yes	4	4
P19	ZH138	65	-	F	newly diagnosed	subtotal	90	no	RT	yes	1	6
P20	ZH139	56	-	F	newly diagnosed	gross total	80	n.d.	TMZ/RT→TMZ	yes	43	52+
P21	ZH148	66	-	M	newly diagnosed	subtotal	60	no	RT	yes	3	6
P22	ZH155	55	-	M	newly diagnosed	partial	60	weakly	RT	yes	2	11
P23	ZH156	81	-	F	newly diagnosed	subtotal	80	no	TMZ	n.d.	3	3+
P24_C	ZH157	46	-	M	newly diagnosed	subtotal	80	no	TMZ/RT→TMZ	yes	5	19

P25	ZH161	55	-	M	newly diagnosed	gross total	90	no	TMZ/RT→TMZ	yes	11	22
P26_D	ZH162	32	-	F	newly diagnosed	subtotal	90	n.d.	RT	yes	9	34+
P27_E	ZH164	67	-	M	newly diagnosed	gross total	90	no	TMZ/RT→TMZ	yes	8	26
P28	ZH165	51	-	M	newly diagnosed	subtotal	70	no	TMZ/RT	yes	2	22
P29	ZH166	63	-	M	newly diagnosed	subtotal	70	n.d.	no therapy	yes	2	2
P30	ZH168	52	-	F	newly diagnosed	gross total	90	n.d.	TMZ/RT→TMZ	yes	6	11
P31	ZH174	34	-	F	newly diagnosed	gross total	80	no	TMZ/RT→TMZ	n.d.	46	46+
P32	ZH180	36	-	F	newly diagnosed	gross total	90	weakly	Bevacizumab + Irinotecan	yes	5	7
P33	ZH182	58	-	M	newly diagnosed	subtotal	90	no	TMZ/RT→TMZ	yes	4	17
P34	ZH188	60	-	F	newly diagnosed	subtotal	60	n.d.	TMZ/RT→TMZ	yes	4	5
P35	ZH194	60	-	F	newly diagnosed	subtotal	70	n.d.	TMZ	yes	11	15
P36	ZH197	42	-	M	newly diagnosed	gross total	90	n.d.	RT	n.d.	12	12+
P37	ZH200	68	-	F	newly diagnosed	gross total	90	no	TMZ/RT→TMZ	yes	5	14
P38	ZH204	60	-	F	newly diagnosed	subtotal	70	n.d.	RT -> TMZ	yes	2	2
P39	ZH205	55	-	M	newly diagnosed	partial	60	no	n.d.	n.d.	1	1+
P40	ZH210	74	-	M	newly diagnosed	subtotal	70	yes	TMZ/RT→TMZ	yes	4	7
P41	ZH221	41	-	F	newly diagnosed	partial	50	weakly	TMZ/RT	yes	3	38
P42	ZH229	66	-	M	newly diagnosed	subtotal	70	no	TMZ/RT→TMZ	yes	4	8
P43	ZH231	55	-	F	newly diagnosed	subtotal	70	no	TMZ/RT→TMZ	yes	6	8
P44	ZH232	37	-	M	newly diagnosed	subtotal	90	no	TMZ/RT→TMZ	yes	3	10+
P45	ZH235	59	-	M	newly diagnosed	subtotal	80	n.d.	no therapy	n.d.	1	1+
P46	ZH241	61	-	F	newly diagnosed	subtotal	90	no	RT -> TMZ	yes	6	14
P47	ZH242	56	-	F	newly diagnosed	subtotal	70	n.d.	TMZ/RT→TMZ	yes	4	5+
P48	ZH250	36	-	F	newly diagnosed	gross total	80	no	TMZ/RT→TMZ	yes	8	15
P49	ZH253	69	-	F	newly diagnosed	gross total	70	n.d.	TMZ/RT→TMZ	yes	6	10+
P50	ZH255	64	-	F	newly diagnosed	gross total	90	yes	TMZ/RT→TMZ	yes	18	31+
P51	ZH256	72	-	F	newly diagnosed	subtotal	80	n.d.	TMZ/RT→TMZ	yes	18	29+
P52	ZH264	61	-	M	newly diagnosed	subtotal	80	n.d.	TMZ/RT→TMZ	yes	4	17
P53	ZH265	38	-	F	newly diagnosed	subtotal	70	n.d.	TMZ/RT→TMZ	yes	5	17
P54	ZH273	35	-	M	newly diagnosed	gross total	80	yes	TMZ/RT→TMZ	yes	22	26+
P55	ZH275	74	-	F	newly diagnosed	subtotal	60	n.d.	RT -> TMZ	yes	2	14

P56	ZH280	85	-	F	newly diagnosed	partial	60	n.d.	no therapy	yes	1	1
P57	ZH283	84	-	F	newly diagnosed	partial	60	n.d.	RT	yes	4	4
P58	ZH303	48	-	F	newly diagnosed	subtotal	90	n.d.	TMZ/RT	yes	3	15+
P59	ZH304	52	-	F	newly diagnosed	subtotal	70	n.d.	TMZ/RT	yes	1	10
P60	ZH315	67	-	M	newly diagnosed	n.d.	n.d.	n.d.	n.d.	n.d.	1	1+
P61	ZH321	72	-	M	newly diagnosed	gross total	80	n.d.	RT	yes	6	16+
P62	ZH326	71	-	M	newly diagnosed	partial	60	n.d.	no therapy	yes	0	0
P63	ZH332	41	-	F	newly diagnosed	subtotal	70	n.d.	TMZ/RT→TMZ	yes	5	18+
P64	ZH333	80	-	M	newly diagnosed	gross total	90	no	RT	yes	10	18+
P65	ZH49	59	60	M	recurrent	gross total	90	yes	TMZ/RT→TMZ	yes	3	18
P66_F	ZH50	18	19	F	recurrent	subtotal	90	n.d.	RT	yes	16	31
P67	ZH61	57	58	M	recurrent	subtotal	90	no	TMZ/RT→TMZ	yes	6	14+
P4_A	ZH86	57	57	F	recurrent	subtotal	70	n.d.	TMZ/RT→TMZ	yes	6	10
P68	ZH87	34	37	M	recurrent	gross total	90	n.d.	RT	yes	5	55
P69	ZH118	59	60	M	recurrent	subtotal	80	n.d.	TMZ/RT→TMZ	yes	10	17
P66_F	ZH124	18	20	F	recurrent	partial	80	n.d.	RT	yes	16	31
P6_B	ZH160	72	74	F	recurrent	gross total	80	n.d.	RT	yes	12	29
P24_C	ZH185	46	47	M	recurrent	subtotal	70	no	TMZ/RT→TMZ	yes	5	19
P70	ZH196	61	62	M	recurrent	subtotal	70	n.d.	TMZ/RT	yes	3	5
P27_E	ZH208	67	68	M	recurrent	subtotal	100	no	TMZ/RT→TMZ	yes	8	26
P71	ZH245	32	34	M	recurrent	subtotal	100	no	TMZ/RT→TMZ	yes	16	17+
P72	ZH258	60	61	M	recurrent	gross total	90	no	TMZ/RT→TMZ	yes	11	22
P73	ZH269	n.d.	59	M	recurrent	n.d.	n.d.	n.d.	n.d.	yes	n.d.	n.d.
P26_D	ZH276	32	34	F	recurrent	gross total	80	n.d.	RT	yes	9	34+
P74	ZH297	39	51	M	recurrent	subtotal	80	n.d.	RT -> TMZ	yes	141	159+

n.d., no data; M, male; F, female; RT, radiotherapy; TMZ, temozolomide; PD, progressive disease; PFS, progression-free survival; OS, overall survival; KPS, Karnofsky performance status; MGMT, O^6-methylguanyl-DNA-methyltransferase, + indicates patients who are deceased; * data are reported from first surgery; ° data are reported from the date of surgery tissue was obtained; A-F indicate patients with two tissue samples in the analysis.

Supplementary Table 3: *Correlation analyses for newly diagnosed patients (upper right) and recurrent patients (lower left).*

	**TGF-β1 mRNA**	**TGF-β_2 mRNA**	**TGF-β_3 mRNA**	**TGF-β_1 protein IHC**	**TGF-β_2 protein IHC**	**TGF-β_3 protein IHC**	**pSmad2 protein IHC**	**pSmad 1/5/8 protein IHC**	**PDGF-B mRNA**	**PAI-1 mRNA**	**PAI-1 protein IHC**	**Ki-67 protein IHC**
**TGF-β_1 mRNA**		r = 0.55<br>p < 0.001<br>***	r = 0.74<br>p < 0.001<br>***	r =0.08<br>p = 0.579<br>n.s.	r = 0.14<br>p = 0.285<br>n.s.	r = -0.03<br>p = 0.818<br>n.s.	r = 0.00<br>p = 0.990<br>n.s.	r = 0.00<br>p = 0.994<br>n.s.	r = 0.78<br>p < 0.001<br>***	r = 0.54<br>p < 0.001<br>***	r = 0.06<br>p = 0.714<br>n.s.	r = -0.19<br>p = 0.146<br>n.s.
**TGF-β_2 mRNA**	r = 0.52<br>p = 0.039<br>*		r = 0.46<br>p < 0.001<br>***	r = 0.06<br>p = 0.682<br>n.s.	r = 0.00<br>p = 0.992<br>n.s.	r = -0.04<br>p = 0.784<br>n.s.	r = -0.01<br>p = 0.940<br>n.s.	r = 0.03<br>p = 0.824<br>n.s.	r = 0.42<br>p < 0.001<br>***	r = 0.39<br>p = 0.001<br>**	r = -0.03<br>p = 0.839<br>n.s.	r = -0.04<br>p = 0.770<br>n.s.
**TGF-β_3 mRNA**	r = 0.83<br>p < 0.001<br>***	r = 0.59<br>p = 0.015<br>*		r = 0.04<br>p = 0.791<br>n.s.	r = -0.02<br>p = 0.897<br>n.s.	r =-0.11<br>p = 0.422<br>n.s.	r = -0.01<br>p = 0.993<br>n.s.	r = -0.13<br>p = 0.351<br>n.s.	r = 0.73<br>p < 0.001<br>***	r = 0.36<br>p = 0.004<br>**	r = -0.18<br>p = 0.254<br>n.s.	r = -0.03<br>p = 0.812<br>n.s.
**TGF-β_1 protein IHC**	r = -0.64<br>p = 0.076<br>n.s.	r = -0.51<br>p = 0.162<br>n.s.	r = -0.61<br>p = 0.086<br>n.s.		r = 0.10<br>p = 0.453<br>n.s.	r = 0.18<br>p = 0.177<br>n.s.	r = 0.11<br>p = 0.413<br>n.s.	r = 0.44<br>p < 0.001<br>***	r = 0.03<br>p = 0.81<br>n.s.	r = 0.17<br>p = 0.213<br>n.s.	r = 0.26<br>p = 0.096<br>n.s.	r = -0.19<br>p = 0.159<br>n.s.
**TGF-β_2 protein IHC**	r = -0.20<br>p = 0.613<br>n.s.	r = -0.27<br>p = 0.493<br>n.s.	r = -0.33<br>p = 0.385<br>n.s.	r = 0.32<br>p = 0.410<br>n.s.		r = 0.22<br>p = 0.099<br>n.s.	r = -0.06<br>p = 0.676<br>n.s.	r = 0.08<br>p = 0.584<br>n.s.	r = 0.14<br>p = 0.293<br>n.s.	r = 0.21<br>p = 0.109<br>n.s.	r = 0.12<br>p = 0.435<br>n.s.	r = -0.17<br>p = 0.207<br>n.s.
**TGF-β_3 protein IHC**	r = -0.38<br>p = 0.313<br>n.s.	r = -0.27<br>p = 0.493<br>n.s.	r = -0.50<br>p = 0.178<br>n.s.	r = 0.29<br>p = 0.437<br>n.s.	r = 0.67<br>p = 0.06<br>n.s.		r = -0.06<br>p = 0.638<br>n.s.	r = 0.16<br>p = 0.253<br>n.s.	r = 0.08<br>p = 0.570<br>n.s.	r = 0.19<br>p = 0.164<br>n.s.	r = 0.07<br>p = 0.659<br>n.s.	r = -0.10<br>p = 0.453<br>n.s.
**pSmad2 protein IHC**	r = -0.49<br>p = 0.155<br>n.s.	r = -0.67<br>p = 0.039<br>*	r = -0.67<br>p = 0.039<br>*	r = 0.71<br>p = 0.037<br>*	r = 0.42<br>p = 0.27<br>n.s.	r = 0.32<br>p = 0.410<br>n.s.		r = 0.14<br>p = 0.306<br>n.s.	r = 0.15<br>p = 0.245<br>n.s.	r = -0.15<br>p = 0.242<br>n.s.	r = 0.17<br>p = 0.264<br>n.s.	r = 0.18<br>p = 0.171<br>n.s.
**pSmad 1/5/8 protein IHC**	r = -0.51<br>p = 0.162<br>n.s.	r = -0.72<br>p = 0.031<br>*	r = -0.51<br>p = 0.162<br>n.s.	r = 0.79<br>p = 0.028<br>*	r = 0.36<br>p = 0.389<br>n.s.	r = 0.43<br>p = 0.299<br>n.s.	r = 0.54<br>p = 0.133<br>n.s.		r = -0.08<br>p = 0.549<br>n.s.	r = 0.01<br>p = 0.962<br>n.s.	r = 0.15<br>p = 0.342<br>n.s.	r = -0.35<br>p = 0.008<br>**
**PDGF-B mRNA**	r = 0.62<br>p = 0.011<br>*	r = 0.32<br>p = 0.226<br>n.s.	r = 0.46<br>p = 0.072<br>n.s.	r = -0.7<br>p = 0.043<br>*	r = 0.32<br>p = 0.410<br>n.s.	r = 0.18<br>p = 0.644<br>n.s.	r = -0.32<br>p = 0.368<br>n.s.	r = -0.51<br>p = 0.162<br>n.s.		r = 0.32<br>p = 0.009<br>**	r = -0.23<br>p = 0.133<br>n.s.	r = -0.16<br>p = 0.228<br>n.s.
**PAI-1 mRNA**	r = 0.54<br>p = 0.033<br>*	r = 0.31<br>p = 0.24<br>n.s.	r = 0.32<br>p = 0.226<br>n.s.	r = -0.02<br>p = 0.982<br>n.s.	r = -0.13<br>p = 0.744<br>n.s.	r = 0.22<br>p = 0.581<br>n.s.	r = 0.33<br>p = 0.349<br>n.s.	r = -0.14<br>p = 0.708<br>n.s.	r = 0.37<br>p = 0.158<br>n.s.		r = 0.32<br>p = 0.036<br>*	r =-0.13<br>p = 0.351<br>n.s.
**PAI-1 protein IHC**	r = 0.3<br>p = 0.498<br>n.s.	r = 0.3<br>p = 0.498<br>n.s.	r = 0.04<br>p = 0.964<br>n.s.	r = -0.04<br>p = 0.964<br>n.s.	r = 0.33<br>p = 0.444<br>n.s.	r = 0.07<br>p = 0.906<br>n.s.	r = 0.3<br>p = 0.498<br>n.s.	r = 0.31<br>p = 0.564<br>n.s.	r = 0.15<br>p = 0.784<br>n.s.	r = 0.3<br>p = 0.498<br>n.s.		r =-0.02<br>p = 0.893<br>n.s.
**Ki-67 protein IHC**	r = 0.20<br>p = 0.613<br>n.s.	r = 0.37<br>p = 0.336<br>n.s.	r = 0.28<br>p = 0.463<br>n.s.	r = 0.21<br>p = 0.619<br>n.s.	r = -0.31<br>p = 0.462<br>n.s.	r = -0.33<br>p = 0.428<br>n.s.	r = -0.20<br>p = 0.613<br>n.s.	r = -0.24<br>p = 0.582<br>n.s.	r = -0.18<br>p = 0.644<br>n.s.	r = -0.52<br>p = 0.236<br>n.s.	r =-0.52<br>p = 0.236<br>n.s.	

Statistical significances of $p < 0.05$ (*), $p < 0.01$ (**) and $p < 0.001$ (***) were determined using the two-tailed Spearman correlation test (r, correlation coefficient). mRNA, messenger ribonucleic acid, IHC, immunohistochemistry, n.s., not significant.

Supplementary Table 4: *TGF-β pathway activity and age.*

	Age
TGF-β_1 mRNA	r=0.15, p=0.240, n.s
TGF-β_2 mRNA	r=0.33, p=0.009, **
TGF-β_3 mRNA	r=0.26, p=0.045, *
TGF-β_1 protein	r=0.03, p=0.817, n.s.
TGF-β_2 protein	r=-0.12, p=0.368, n.s.
TGF-β_3 protein	r=-0.14, p=0.314, n.s.
pSmad2 protein	r=0.06, p=0.661, n.s.
pSmad1/5/8 protein	r=-0.17, p=0.204, n.s.
PDGF-B mRNA	r=0.09, p=0.472, n.s.
PAI-1 mRNA	r=0.26, p=0.044, *
PAI-1 protein	r=-0.1, p=0.524, n.s.
Ki-67 protein	r=0.09, p=0.492, n.s.

Correlation analyses were performed in the group of newly diagnosed glioblastoma patients. Statistical significances of p<0.05 (*) and p<0.01 (**) were determined using the two-tailed Spearman correlation test (r, correlation coefficient). mRNA, messenger ribonucleic acid, n.s., not significant.

SUPPLEMENTARY FIGURES

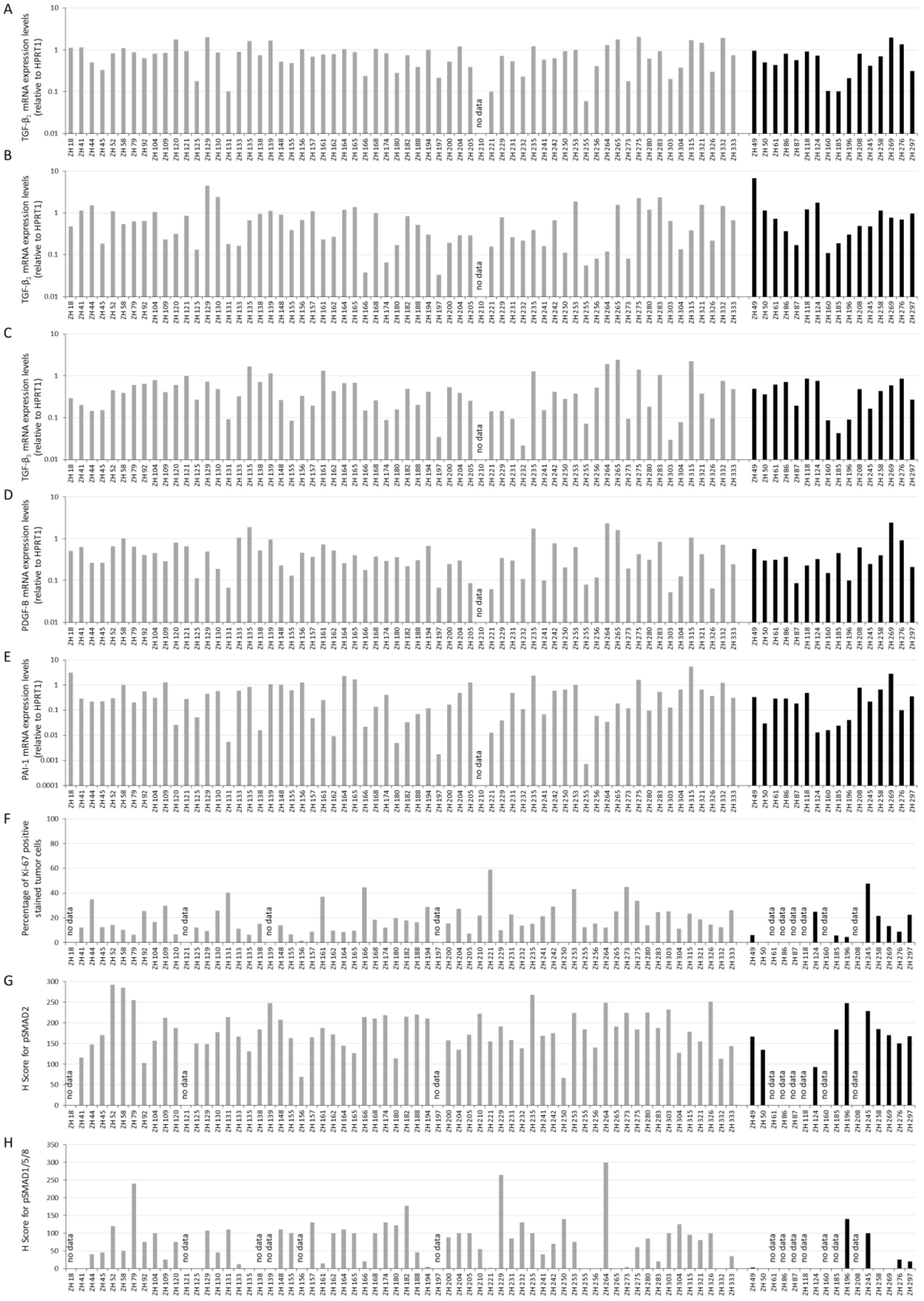

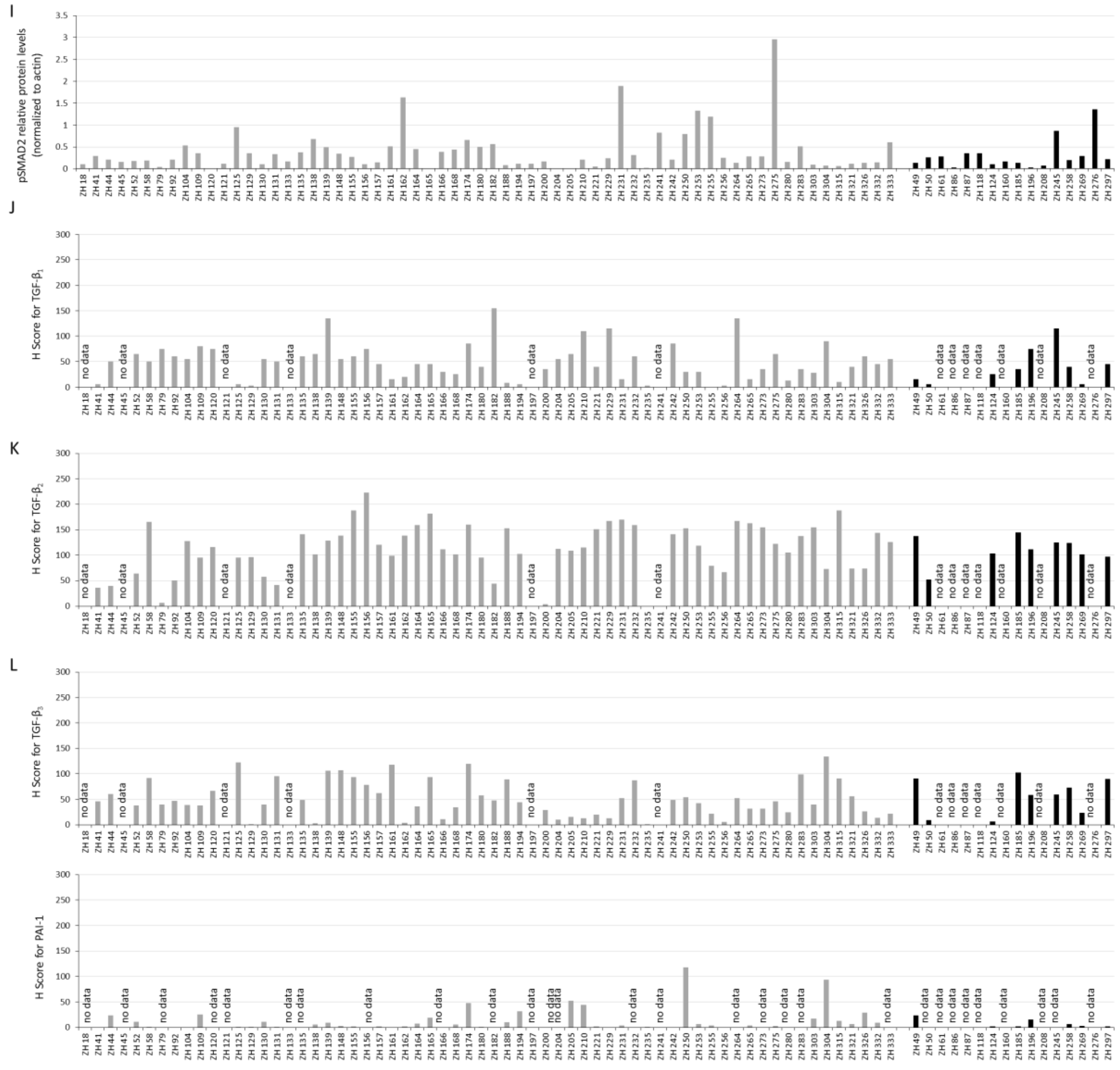

Supplementary Figure 1: *TGF-β-related candidate biomarker determination in glioblastoma tissue.* Relative mRNA expression levels were assessed for TGF-β_1 (A), TGF-β_2 (B), TGF-β_3 (C), PDGF-B (D) and PAI-1 (E) using hypoxanthine-guanine phosphoribosyltransferase1 (HPRT1) as a reference. Values are represented in a logarithmic scale. Grey bars show newly diagnosed and black bars recurrent glioblastoma patients. Protein levels were assessed by immunohistochemistry. The percentage of positive tumor cells was assessed for Ki-67 (F) and H scores were assessed for TGF-β_1 (G), TGF-β_2 (H), TGF-β_3 (I), pSmad2 (J), pSmad1/5/8 (K) and PAI-1 (L). Grey bars show newly diagnosed, black bars show recurrent glioblastoma patients

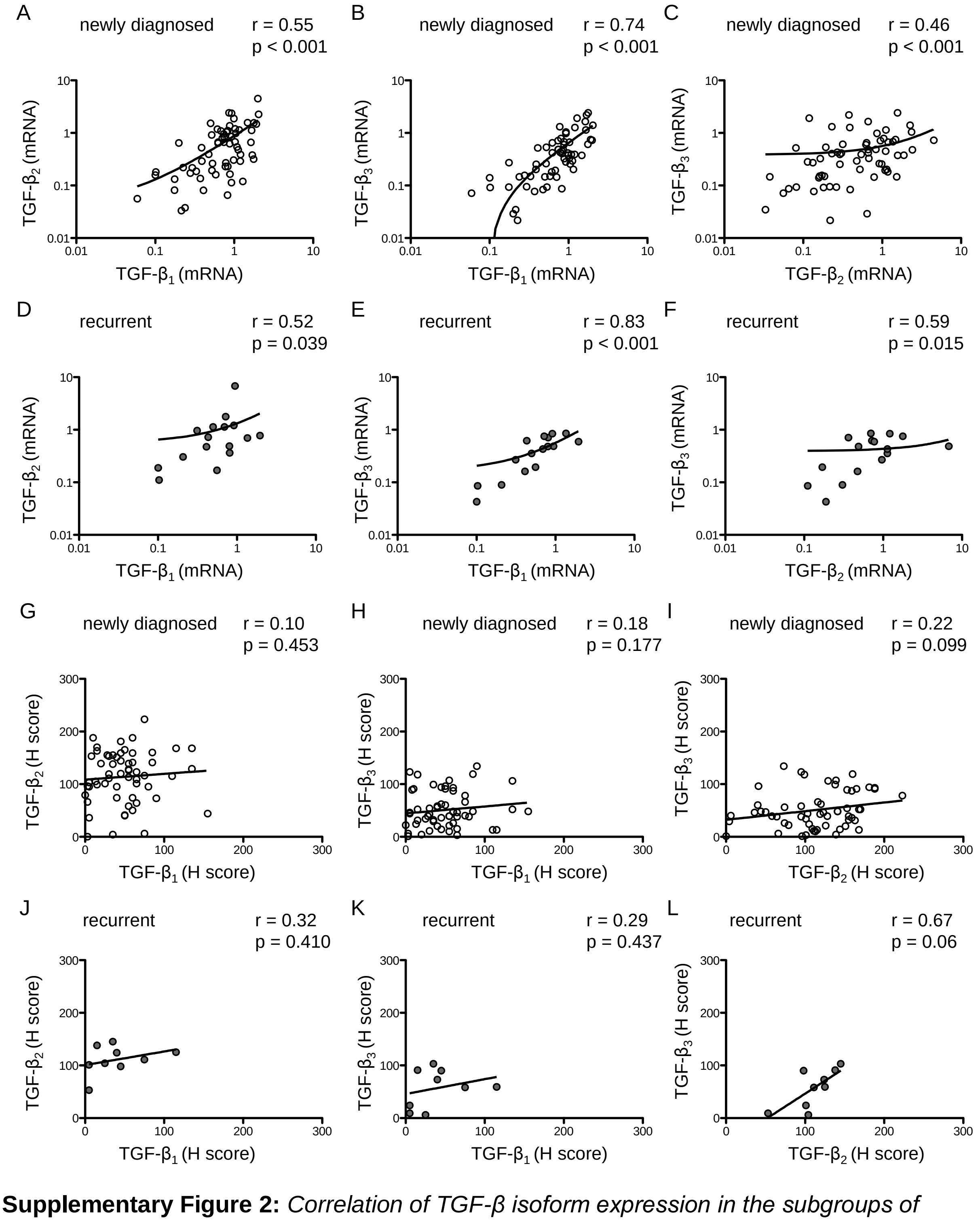

Supplementary Figure 2: *Correlation of TGF-β isoform expression in the subgroups of newly diagnosed or recurrent glioblastomas.*

Correlation of mRNA data of TGF-β_1 and TGF-β_2 (A,D), of TGF-β_1 and TGF-β_3 (B,E) and of TGF-β_2 and TGF-β_3 (C,F) is shown for the patient subgroups of newly diagnosed patients (A-C) and recurrent patients (D-F). Values are represented in a logarithmic scale. Correlation of protein data of TGF-β_1 and TGF-β_2 (G,J), of TGF-β_1 and TGF-β_3 (H,K) and of TGF-β_2 and TGF-β_3 (I,L) is shown for the patient subgroups of newly diagnosed patients (G-I) and recurrent patients (J-L). Values are represented in a linear scale. Two-tailed Spearman test coefficients (r) and significances (p) are indicated (open circles, newly diagnosed; closed circles, recurrent tumor specimens).

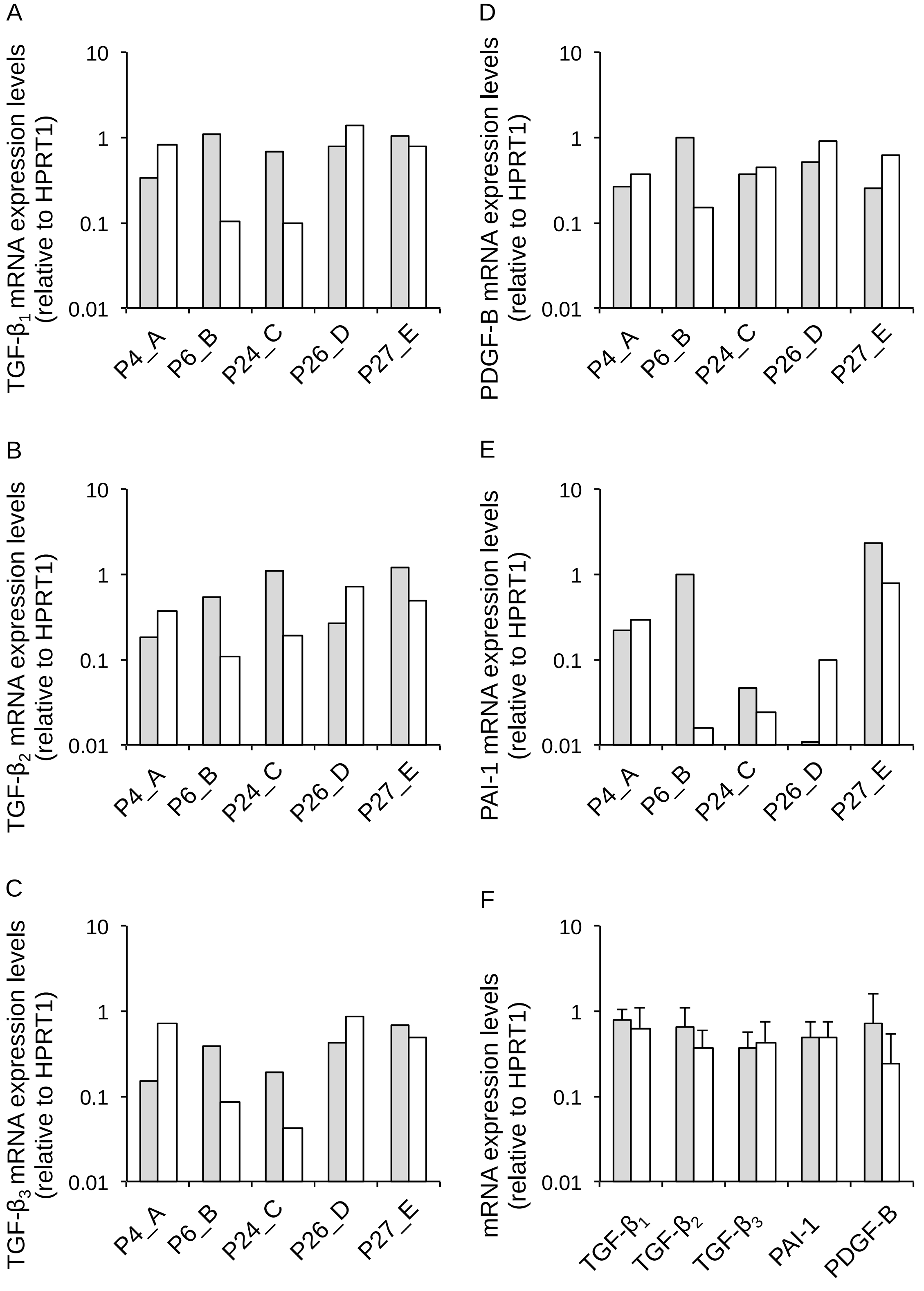

Supplementary Figure 3: *mRNA expression in paired glioblastoma (newly diagnosed and recurrent).* TGF-β_1 (A), TGF-β_2 (B),TGF-β_3 (C), PDGF-B (D) and PAI-1 (E) mRNA expression levels are shown for primary (grey bars) and recurrent (white bars) tumor specimens of 5 individual glioblastoma patients. Values are represented in a logarithmic scale. F, Pooled mRNA expression levels of the five patients are shown for the target genes. Analysis of significance was carried out using the two-tailed Student's *t*-test. Patient tissues of primary and recurrent tumor: P4_A (ZH 45 and ZH 86), P6_B (ZH 58 and ZH 160), P24_C (ZH 157 and ZH 185), P26_D (ZH 162 and ZH 276), P27_E (ZH 164 and ZH 208).

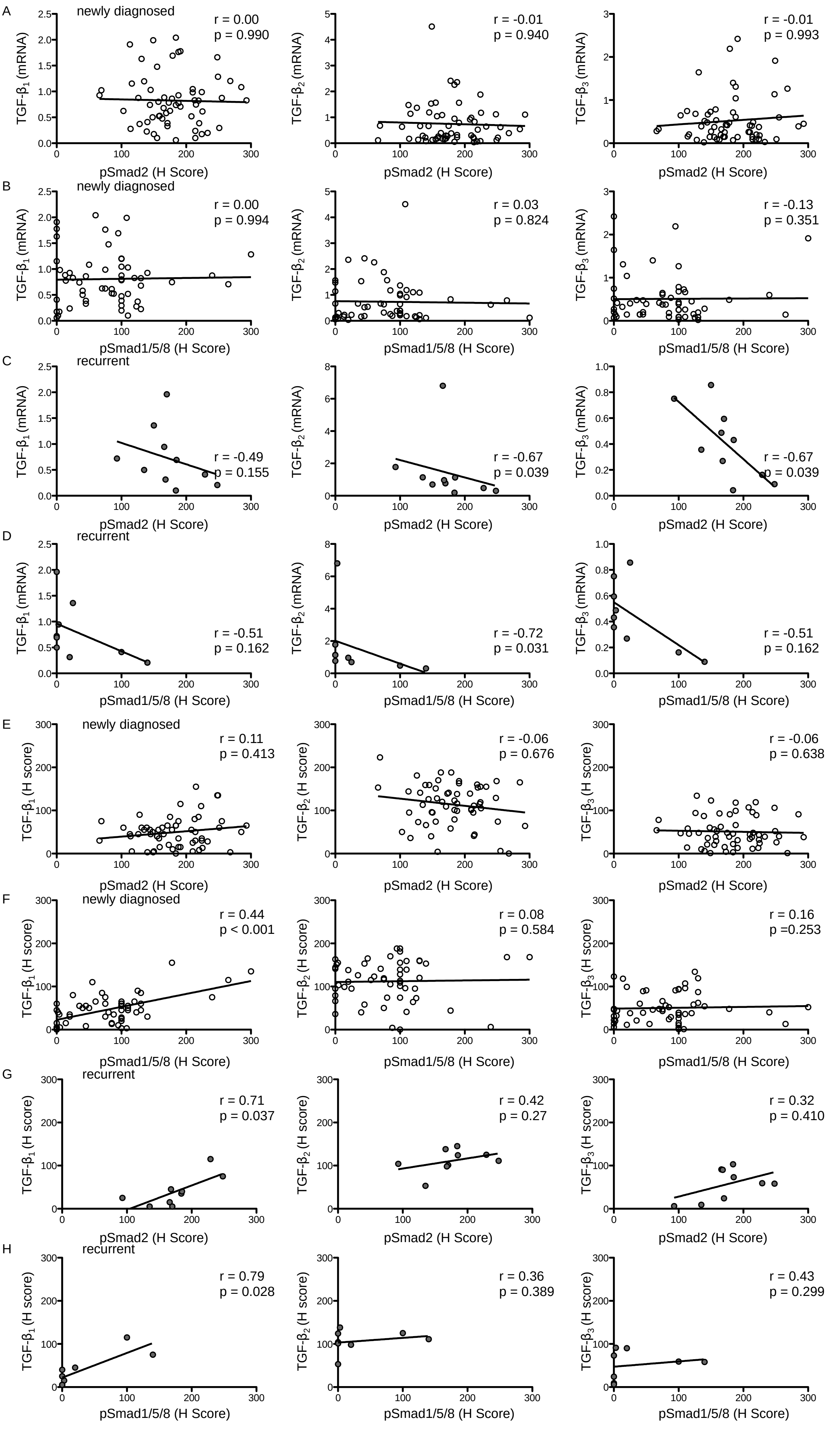
A
newly diagnosed
r = 0.00
p = 0.990
r = -0.01
p = 0.940
r = -0.01
p = 0.993
TGF-β1 (mRNA)
TGF-β2 (mRNA)
TGF-β3 (mRNA)
pSmad2 (H Score)
B
newly diagnosed
r = 0.00
p = 0.994
r = 0.03
p = 0.824
r = -0.13
p = 0.351
pSmad1/5/8 (H Score)
C
recurrent
r = -0.49
p = 0.155
r = -0.67
p = 0.039
r = -0.67
p = 0.039
D
recurrent
r = -0.51
p = 0.162
r = -0.72
p = 0.031
r = -0.51
p = 0.162
E
newly diagnosed
r = 0.11
p = 0.413
r = -0.06
p = 0.676
r = -0.06
p = 0.638
TGF-β1 (H score)
TGF-β2 (H score)
TGF-β3 (H score)
F
newly diagnosed
r = 0.44
p < 0.001
r = 0.08
p = 0.584
r = 0.16
p =0.253
G
recurrent
r = 0.71
p = 0.037
r = 0.42
p = 0.27
r = 0.32
p = 0.410
H
recurrent
r = 0.79
p = 0.028
r = 0.36
p = 0.389
r = 0.43
p = 0.299

Supplementary Figure 4: *Assessment of TGF-β pathway activation in glioblastoma: Smad phosphorylation in the subgroups of newly diagnosed or recurrent glioblastomas.*
A-D, Correlation of mRNA data of TGF-β_1 or TGF-β_2 or TGF-β_3 and pSmad2 (A,C) or pSmad1/5/8 (B,D) for newly diagnosed patients (A,B) or recurrent patients (C,D). E-H, Correlation of protein data of TGF-β_1 or TGF-β_2 or TGF-β_3 and pSmad2 (E,G) or pSmad1/5/8 (F,H) for newly diagnosed patients (E,F) or recurrent patients (G,H). Values are represented in a linear scale. Two-tailed Spearman test coefficients (r) and significances (p) are indicated (open circles, newly diagnosed; closed circles, recurrent tumor specimens).

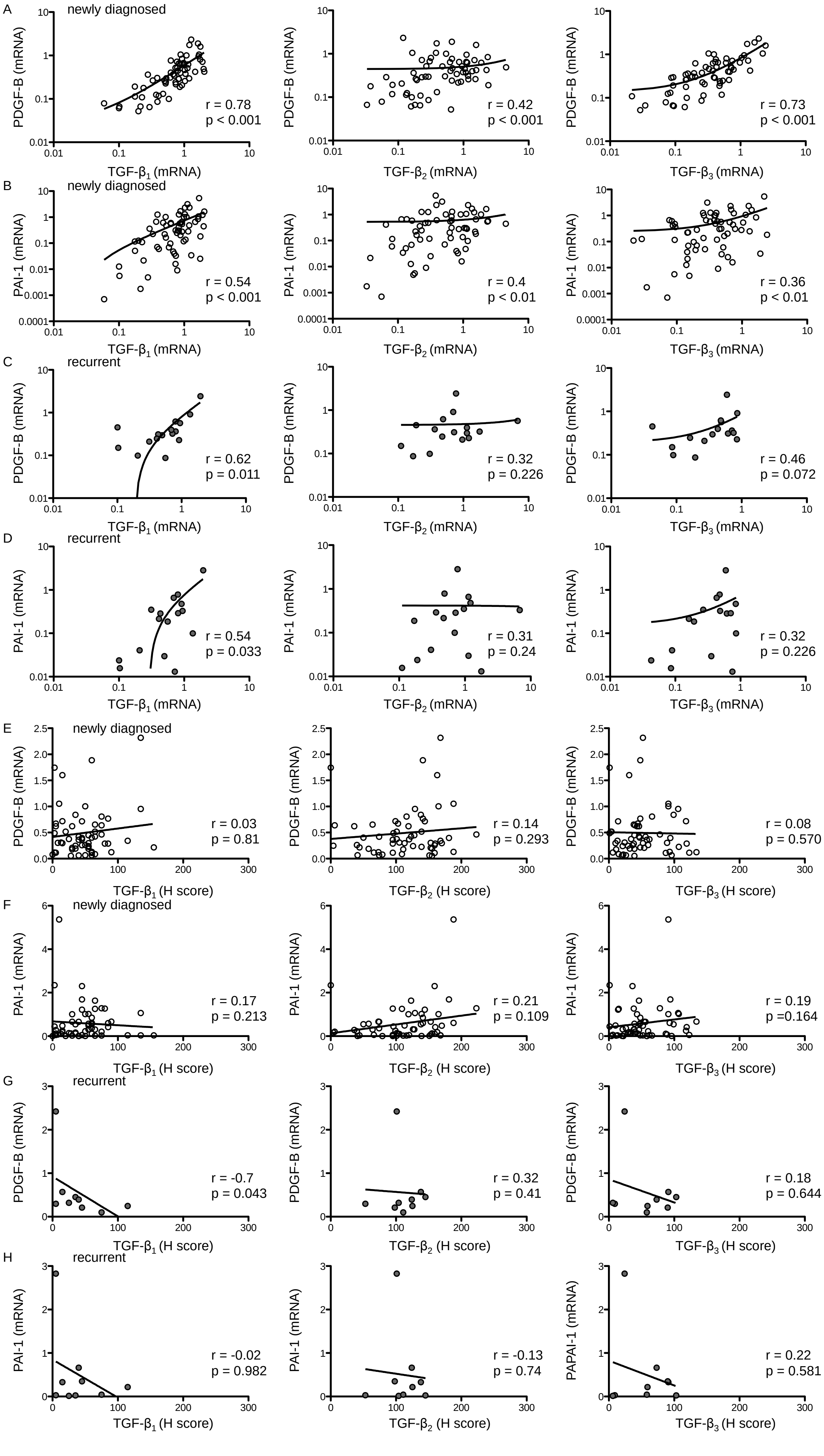
A
newly diagnosed
PDGF-B (mRNA)
TGF-β1 (mRNA)
r = 0.78
p < 0.001
TGF-β2 (mRNA)
r = 0.42
p < 0.001
TGF-β3 (mRNA)
r = 0.73
p < 0.001
B
newly diagnosed
PAI-1 (mRNA)
TGF-β1 (mRNA)
r = 0.54
p < 0.001
TGF-β2 (mRNA)
r = 0.4
p < 0.01
TGF-β3 (mRNA)
r = 0.36
p < 0.01
C
recurrent
PDGF-B (mRNA)
TGF-β1 (mRNA)
r = 0.62
p = 0.011
TGF-β2 (mRNA)
r = 0.32
p = 0.226
TGF-β3 (mRNA)
r = 0.46
p = 0.072
D
recurrent
PAI-1 (mRNA)
TGF-β1 (mRNA)
r = 0.54
p = 0.033
TGF-β2 (mRNA)
r = 0.31
p = 0.24
TGF-β3 (mRNA)
r = 0.32
p = 0.226
E
newly diagnosed
PDGF-B (mRNA)
TGF-β1 (H score)
r = 0.03
p = 0.81
TGF-β2 (H score)
r = 0.14
p = 0.293
TGF-β3 (H score)
r = 0.08
p = 0.570
F
newly diagnosed
PAI-1 (mRNA)
TGF-β1 (H score)
r = 0.17
p = 0.213
TGF-β2 (H score)
r = 0.21
p = 0.109
TGF-β3 (H score)
r = 0.19
p =0.164
G
recurrent
PDGF-B (mRNA)
TGF-β1 (H score)
r = -0.7
p = 0.043
TGF-β2 (H score)
r = 0.32
p = 0.41
TGF-β3 (H score)
r = 0.18
p = 0.644
H
recurrent
PAI-1 (mRNA)
TGF-β1 (H score)
r = -0.02
p = 0.982
TGF-β2 (H score)
r = -0.13
p = 0.74
PAPAI-1 (mRNA)
TGF-β3 (H score)
r = 0.22
p = 0.581

Supplementary Figure 5: *Assessment of TGF-β pathway activation in glioblastoma: expression of TGF-β response genes in the subgroups of newly diagnosed or recurrent glioblastomas.*
A-D, Correlation of mRNA data of TGF-$β_1$ or TGF-$β_2$ or TGF-$β_3$ and PDGF-B (A,C) or PAI-1 (B,D) for newly diagnosed patients (A,B) or recurrent patients (C,D). Values are represented in a logarithmic scale. E-H, Correlation of protein data of TGF-$β_1$ or TGF-$β_2$ or TGF-$β_3$ and PDGF-B (E,G) or PAI-1 (F,H) for newly diagnosed patients (E,F) or recurrent patients (G,H). Values are represented in a linear scale. Two-tailed Spearman test coefficients (r) and significances (p) are indicated (open circles, newly diagnosed; closed circles, recurrent tumor specimens).

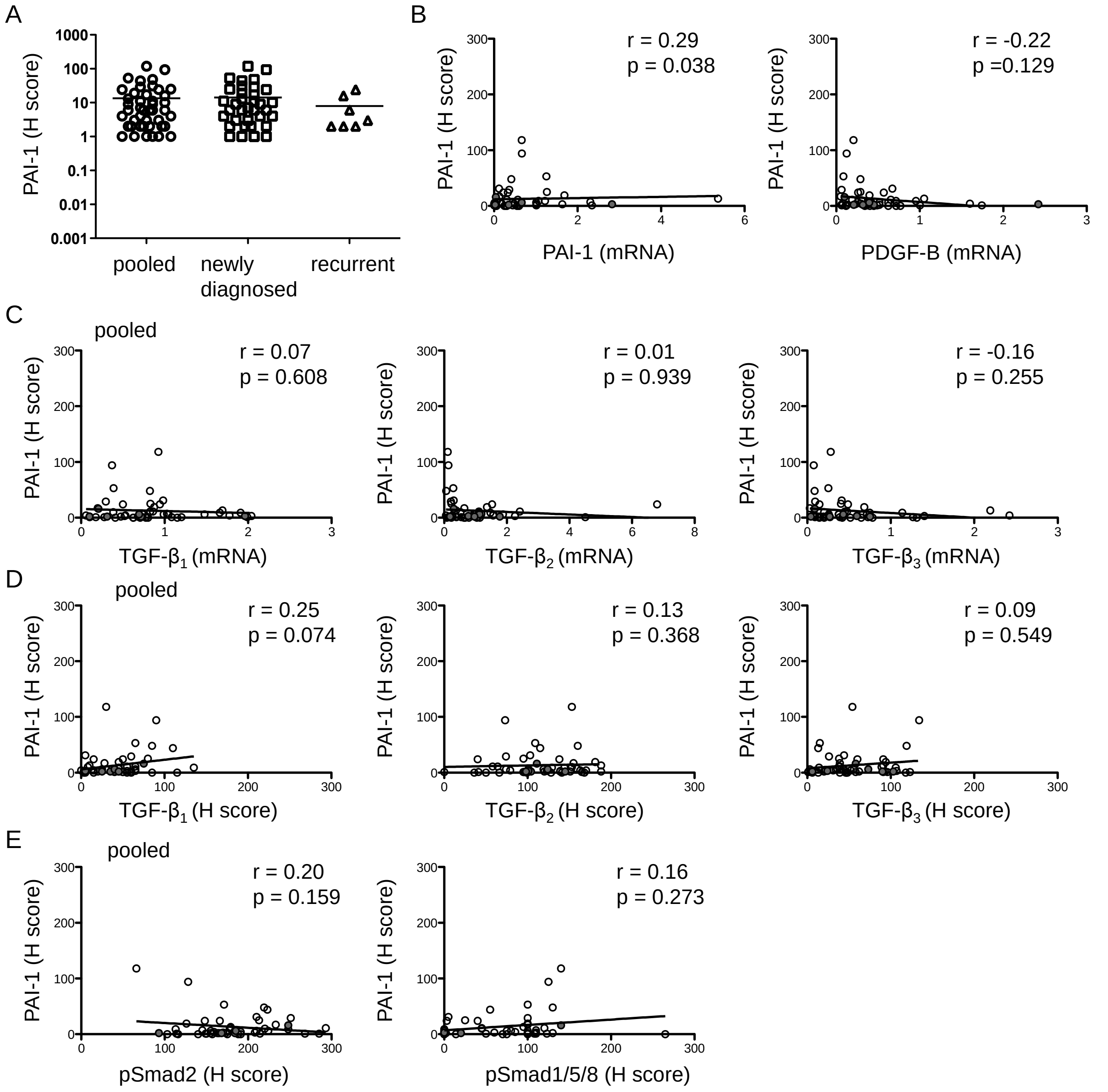

Supplementary Figure 6: *Assessment of TGF-β pathway activation in glioblastoma: PAI-1 protein levels.*

A, PAI-1 protein levels were assessed by immunohistochemistry and median H Scores are shown for all patients pooled, newly diagnosed tumor tissues or recurrent tumor tissues separately. The black bar marks the mean in each group. Correlation is shown for the H scores of PAI-1 and mRNA expression levels of PAI-1 or PDGF-B (B) for all samples pooled. (B) Pairwise correlation analyses of PAI-1 protein levels and TGF-β isoform mRNA (C) or protein (D) levels are shown for all samples pooled. Correlation is shown for the H scores of PAI-1 and pSmad2 or pSmad1/5/8 (E) for all samples pooled. Two-tailed Spearman test coefficients (r) and significances (p) are indicated (open circles, newly diagnosed; closed circles, recurrent).

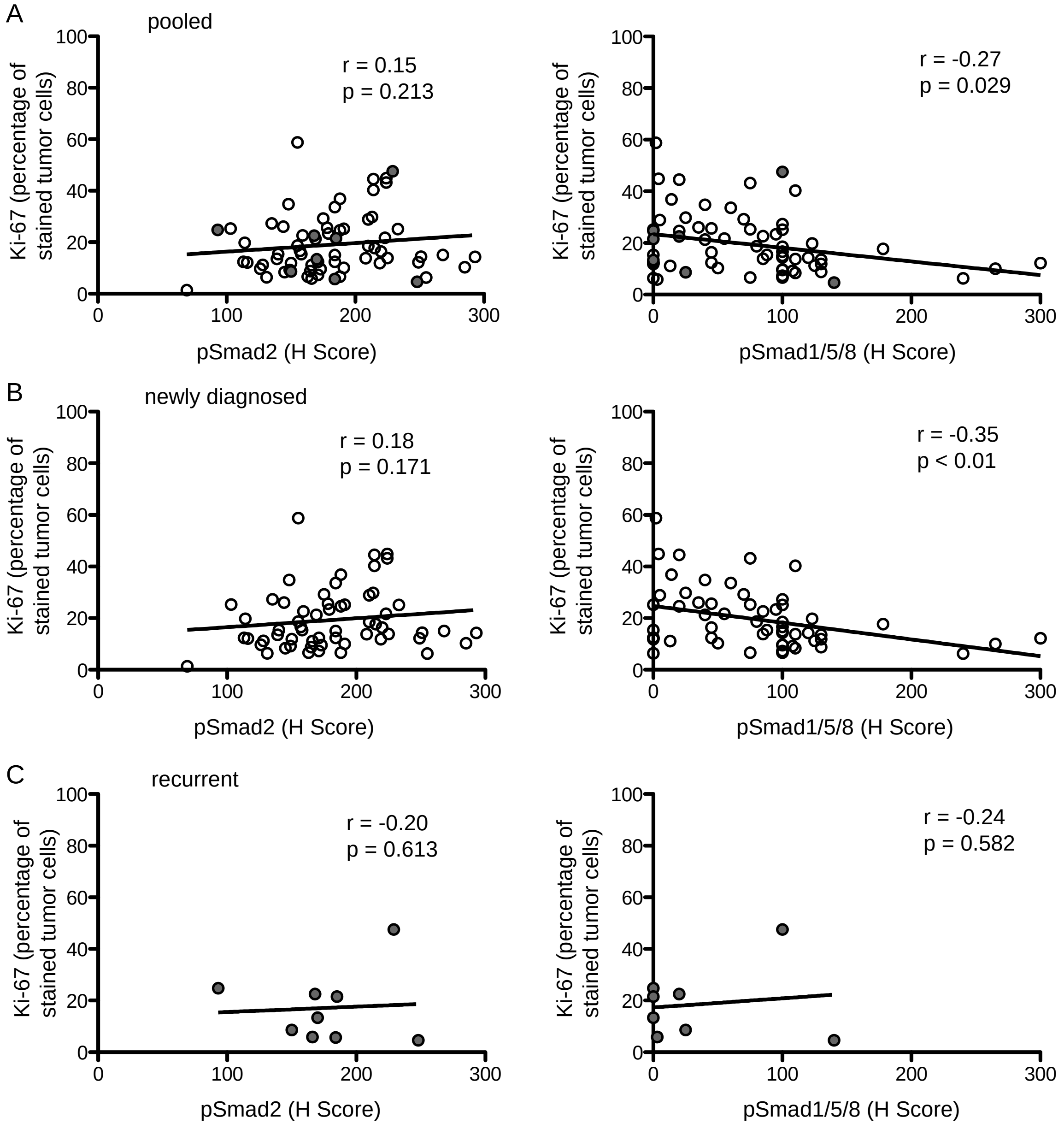

Supplementary Figure 7: *Correlation of pSmad2 or pSmad1/5/8 and Ki-67.*
Correlation analyses of protein data of pSmad2 or pSmad1/5/8 and Ki-67 were assessed for all patients pooled (A), newly diagnosed patients (B) or recurrent patients (C). Values are represented in a linear scale. Two-tailed Spearman test coefficients (r) and significances (p) are indicated (open circles, newly diagnosed; closed circles, recurrent tumor specimens).

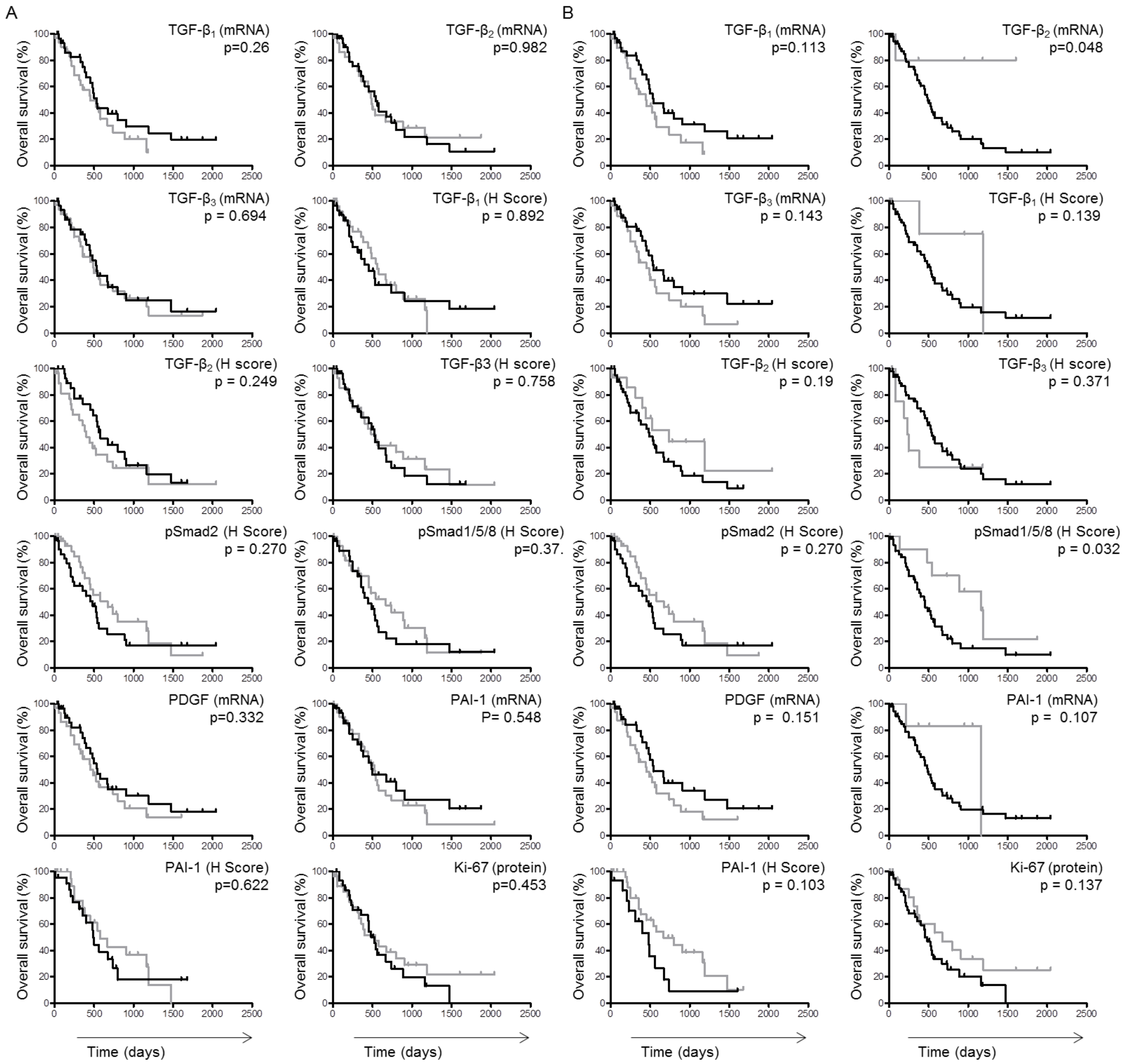

Supplementary Figure 8: *TGF-β pathway activity and outcome.* Kaplan-Meier survival curves of overall survival are shown for newly diagnosed glioblastomas. Patients were divided into two groups with high (black) or low (grey) expression of the target gene or target protein. The cut-off was defined by the median mRNA expression level of the target (A) or the expression level that results in the highest impact on survival (B). Statistical significances (p) were determined using the log-rank test ($p < 0.05$ was considered significant).

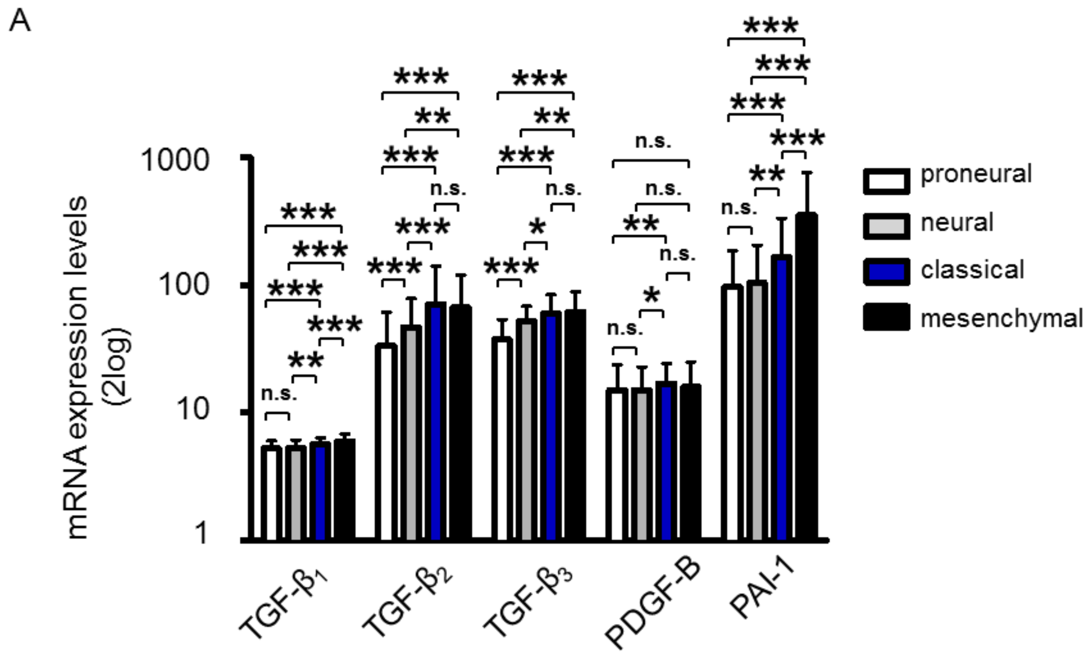

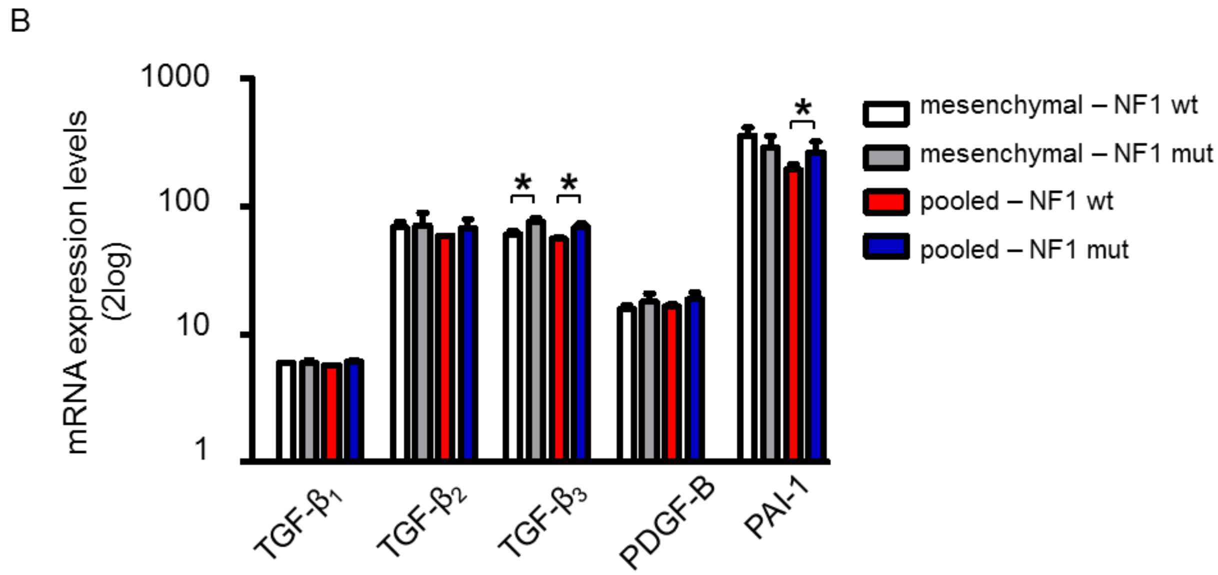

Supplementary Figure 9: *TGF-$\beta_{1\text{-}3}$ gene and TGF-β target gene expression in glioblastoma subtypes based on gene expression data.* A, mRNA expression levels are shown for proneural, neural, classical and mesenchymal glioblastoma patients. B, mRNA expression levels are shown for glioblastoma patients based on their NF1 mutation status. Statistical significances of $p < 0.05$ (*), $p < 0.01$ (**) and $p < 0.001$ (***) were determined using the Mann-Whitney test. Values are represented in a logarithmic scale. Data were obtained from the TCGA database.

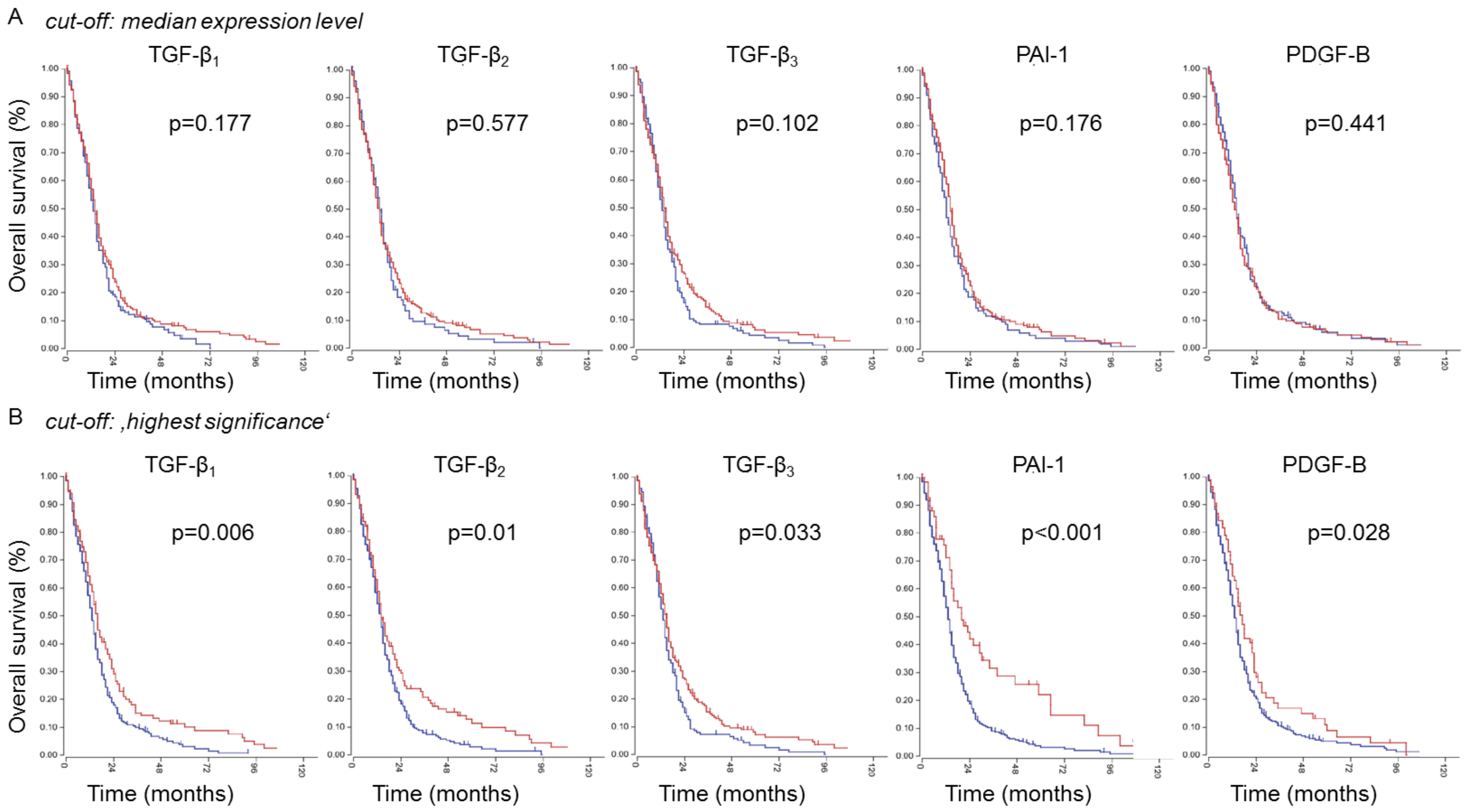

Supplementary Figure 10: *TGF-β pathway activity and outcome – an analysis of the TCGA database.*
Kaplan-Meier survival curves of overall survival are shown for newly diagnosed glioblastomas. Patients were divided into two groups with high (blue) or low (red) expression of the target gene. The cut-off was defined by the median mRNA expression level of the target (A) or the expression level that results in the highest association with survival (B). Statistical significances (p) were determined using the log-rank test ($p < 0.05$ was considered significant).
